# Measurement-Based Optimization of a Lightweight Upper-Extremity Rehabilitation Exoskeleton for Task-Oriented Treatment

**DOI:** 10.3390/s26061849

**Published:** 2026-03-15

**Authors:** Piotr Falkowski, Piotr Kołodziejski, Krzysztof Zawalski, Maciej Pikuliński, Jan Oleksiuk, Tomasz Osiak, Andrzej Zakręcki, Kajetan Jeznach, Daniel Śliż

**Affiliations:** 1 Łukasiewicz Research Network—Industrial Research Institute for Automation and Measurements PIAP, 02-486 Warsaw, Poland; 2 Institute of Aeronautics and Applied Mechanics, Warsaw University of Technology, 00-661 Warszawa, Poland; 3 Department of Biocybernetics and Biomedical Engineering, Faculty of Electrical Engineering, Automatics, Computer Science and Biomedical Engineering, AGH University of Science and Technology in Cracow, 30-059 Kraków, Poland; 4 3rd Department of Cardiology and Internal Medicine, Warsaw Medical University, 02-091 Warszawa, Poland

**Keywords:** exoskeleton, digital shadow, finite element method, multibody system dynamics, parametric optimization, rehabilitation robotics, simulation-based design, topology optimization

## Abstract

Contemporary physiotherapy requires technological tools to provide effective therapy to the increasing group of patients with neurological conditions, among others. This can be achieved with rehabilitation robots, which can also be exoskeletons—wearable devices that mobilize multiple joints with complex motions representing activities of daily living. To perform kinesiotherapy conveniently in home-like environments, the exoskeletons need to be relatively lightweight. The paper presents the methodology for decreasing the mass of the exoskeleton design with real-life data-driven simulations of motions, followed by multibody dynamics simulations, and finite element method (FEM) multistep optimization. The process includes sequential initial parametric optimization, topology optimization, and final parametric optimization. The steps are used to set initial dimensional and material parameters, extract new geometrical features, and adjust the final geometry dimensions of a new design. The presented case of the *SmartEx-Home* exoskeleton resulted in a total mass reduction of almost 50% for the main construction elements while meeting the criteria of the minimum safety factor and maximum internal stress and strain for all components. The final design was manufactured and tested with humans, reflecting an almost fully automatic passive and active therapy.

## 1. Introduction

Impaired upper limb function greatly affects activities of daily living, which form the basis for independent functioning [1,2]. Upper limb disability resulting from musculoskeletal system dysfunction constitutes a significant health issue both in the general population and among patients receiving primary healthcare services. The prevalence of upper limb disorders varies considerably, ranging from 2% to 53%, depending on the study, with higher incidence rates observed among students and professionally active individuals. Musculoskeletal complaints affecting the upper limb are associated with specific pathological conditions, such as rotator cuff tendinitis, adhesive capsulitis, lateral epicondylitis, carpal tunnel syndrome, and De Quervain’s disease. In some cases, these disorders present with nonspecific symptoms, making their classification and diagnosis more challenging [3].

It is not only orthopedic conditions that cause upper limb impairment. Many neurological diseases can also cause upper limb impairment [4]. For example, in the United States alone, more than 795,000 people have a stroke each year [4,5]. Stroke-related costs in the United States totalled nearly $56.2 billion between 2019 and 2020. These costs include the cost of health care services, medicines to treat stroke, and missed days of work [6]. Among the numerous neurological deficits that occur in post-stroke patients, loss of upper limb motor function is the most common, affecting 77.4% of patients. This impairment persists for more than six months in 89% of those who have experienced loss of upper limb function, significantly affecting their quality of life. The process of neuroplasticity enables partial or full recovery of function, and its effectiveness can be enhanced by intensive and repetitive motor activity as part of rehabilitation therapy [7]. Another disease that can be mentioned is multiple sclerosis. The number of people affected by multiple sclerosis worldwide was estimated at 2.8 million in 2020. Using the same methodology as in 2013, this number was estimated to be 30% higher than the 2013 estimate. The global prevalence in 2020 was 35.9 cases per 100,000 people [8], and 60% of people with multiple sclerosis have impaired hand function. Upper limb dysfunction in activities of daily living is greater than that observed after stroke, as both sides are often affected [4]. In neurological diseases, impairment of the upper limbs may result from various causes. In neuromuscular diseases such as spinal muscular atrophy, there are also disorders of the upper limbs resulting from muscle weakness [9]. The above examples are selected neurological diseases causing impairment of the upper limb, but there are more [2,4,10]

Among the tools that significantly support the physiotherapy process, especially considering medical staff shortages, are robots [11]. Those used in the home environment must address many challenges, including safety, cost, environmental requirements, and use-independent behavior [12]. Many patients need continuous therapy at home after returning from the hospital, which can bring additional benefits. Post-stroke individuals feel comfortable in a home environment because it is more familiar and closer to family and friends. Providing home therapy results in a lower likelihood of the patient’s re-hospitalization, which relieves the burden on the healthcare system. Home rehabilitation removes access barriers for people who have problems with mobility and travel. In addition, it allows work during pandemic conditions when restrictions on physical contact are imposed; robots for home rehabilitation are especially useful in such situations. Home rehabilitation requires a high degree of repetition for the activities being taught to be effective. Automated therapy sessions can enable increased repetitions of rehabilitation exercises without the need to intensify in-person visits while providing patients with engaging and goal-oriented activities to support the therapy process [7].

Rehabilitation robots are either end-effector types or exoskeletons [13]. The former enable only point interaction between the device and the human. However, this makes these robots more universal and easier to use from a safety perspective [13]. In contrast, the latter provide more complex interaction, including mobilization of particular joints [14,15]. However, they are significantly susceptible to mechanical differences between the exoskeleton’s kinematic chain and the anatomy of an individual user. Due to the advantages of controlling every degree of freedom precisely with exoskeletons, these structures can be particularly useful for telerehabilitation [12,16].

As at least partially wearable structures, exoskeletons benefit from lightweight construction [11,17]. To achieve the lowest possible mass while preserving the required durability and strength, optimization methods are utilized during the design process.

One of the commonly used methods is parametric optimization (PO). It requires defining all possible parameters and their ranges, and is often quite complicated to perform. However, it allows for a more thorough overview of the process and leaves more decisions to the user. The PO process is commonly reinforced by sensitivity analysis (SA) and the response surface method (RSM) to help reduce the overall time of the optimization [18].

SA is a method used to determine how changes in the outputs of a mathematical model or function are affected by changes in the input parameters [19]. It is common practice to use it to determine the most suitable parameter configuration and reduce the number of input parameters, thereby reducing the overall time of the optimization process.

Another tool used to optimize designs is topology optimization (TO) [20]. It is a mathematical method that spatially optimizes the distribution of material within a defined domain while maintaining previously set constraints and looking for the minimum value of the predefined objective function [21]. It is used in the structural optimization of various parts, from mechanical parts such as gears or wheels to smaller, more precise objects such as ferrite plates [22]. The method was used in the literature to optimize rehabilitation exoskeletons [23]. Such an approach may utilize different methods like the solid isotropic material with penalization (SIMP) method [24], evolutionary structural optimization (ESO) [25], genetic algorithms [26], or others. The SIMP method is based on the finite element method (FEM) analysis and is commonly applied in stress and structure durability research [24].

The presented methods are based on different approaches but can be used for the same purpose. They can also be applied to additively manufactured components [27]. Moreover, they can be used sequentially to obtain even higher mass reduction [28]. For this reason, the paper focuses on combining PO and TO techniques for a specific application in rehabilitation exoskeleton design, using a series robot as an example.

**Figure 1:**
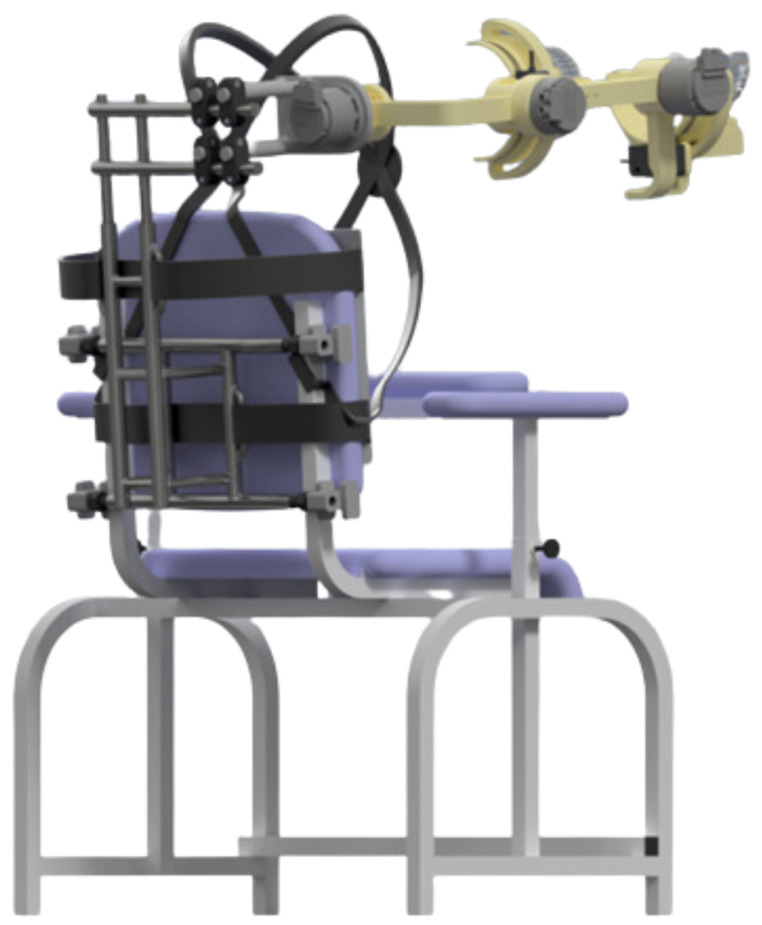
Visual ofthe SmartEx-Home exoskeleton assembled to the chair.

The exoskeleton that is optimized in this paper is *SmartEx-Home*, a device for task-oriented kinesiotherapy of the upper extremity. It can be attached to a chair, a bed, or used on a dedicated frame with a lifting column. Therefore, physiotherapy can be performed in a sitting, lying, or standing position (see Figures S1–S3). The device enables motion in five degrees of freedom at the shoulder and elbow joints. Therefore, it is suitable for task-oriented exercises. Shoulder abduction/adduction and flexion/extension, as well as elbow flexion/extension, are driven by electric motors. In contrast, shoulder and ulnar-radial rotations are provided by the passive joints of the exoskeleton. The construction is adjustable to the lengths of arm and forearm segments corresponding to the Polish population from the 5th percentile for women to the 95th percentile for men [29]. Therefore, all of the joint rotation axes can be aligned with the modeled rotation axes of human joints. The main segments are attached to the arm and forearm through 3-axis force sensors with elastic braces. Patients can also be secured for exercise with wheelchair belts. Exercises with the device are performed in either passive or active mode. Their trajectories correspond to the activities of daily living modeled and recorded in a previous stage by physiotherapists [30]. A visual of the exoskeleton attached to the chair is shown in Figure 1.

Based on an analysis of recent research papers regarding designs of rehabilitation devices, combining topology optimization with parametric optimization based on real-life pre-measured motions has not yet been widely investigated and applied [16]. Therefore, there is a research gap in the field of optimizing rehabilitation or assistive robots, which have complex loads resulting from human-machine physical interactions and dynamic motions. The focus of the study is to apply the methodology in the field of biorobotics and validate possible mass minimization while modeling reactions using real-life motion recordings.

The aim of the study is to minimize the mass of the *SmartEx-Home* exoskeleton [30,31] using numerical methods. These include combined multibody dynamics simulations and multistep FEM optimization. The process is based on real-life measured motions used in planned robot-aided physiotherapy. Therefore, the optimization process is dedicated to the specific application of the device. Currently, the methods that are used tend to involve only a single type of optimization and are based on generalized loads, not specific therapy motions. Therefore, the resultant designs cannot be treated as optimal. The presented methodology will fill this gap. To clearly highlight the novelty of the proposed engineering approach, the main contributions of this study are summarized as follows:

- A measurement-based optimization framework integrating real-life motion recordings of functional exercises reflecting activities of daily living into the design process of a rehabilitation exoskeleton, ensuring that load cases reflect actual therapy conditions rather than generalized assumptions;
- A combined multistep optimization strategy that sequentially integrates multibody dynamics (MBD), initial parametric optimization, topology optimization, and final parametric optimization;
- A design methodology enabling significant mass reduction (by approximately 50% for the structural components) while maintaining safety factors suitable for medical devices;
- Verification of the optimized design through physical prototyping and experimental testing with human participants.

Moreover, this paper will present the complex simulation-based design and discuss the effects obtained at every stage.

## 2. General Methodology

Optimization was divided into six main stages presented in Table 1. The initial design presented in Figure 1 was created based on the anthropometric modeling of the human upper extremity to suit people between the 5th percentile for women and the 95th percentile for men of the Polish population [29]. It aimed to provide full mobility of the shoulder and elbow joints. During the initial design, it was assumed that the driven shafts needed to be bearing-mounted and that all attachments to the user’s body needed to be adjustable so that the anatomical axes of the extremity would overlap with the rotational axes of the device. The initial design was manufactured as part of a prior research project and served as a benchmark for the optimization.

Then, the set of motions useful for task-oriented kinesiotherapy was defined, extending sets previously used in analogical studies [32,33]. These motions were recorded with the use of five inertial measurement units (IMUs; one reference and four attached to the body) and processed to represent joint variables for the exoskeleton model [31]. These were then used as inputs for the inverse dynamics simulation to calculate resultant forces and torques at the characteristic points of the exoskeleton. These points include the interfaces between moving segments of the device and attachments of the exoskeleton acting upon the extremity. The parameters obtained at this stage were used for further finite element method simulations.

The finite element computations consisted of initial strength validation with potential design improvements, initial parametric optimization to adjust the overall dimensions before the main mass reduction, followed by topology optimization to extract new features that reduce the total mass, and final parametric optimization for the final fine-tuning of the extracted features. All stages except the last one were based on an isotropic material model, which was sufficient at these stages for components manufactured by the selective laser sintering (SLS) method [34]. However, the last stage was performed with an orthotropic model for the most accurate results.

**Figure 2:**
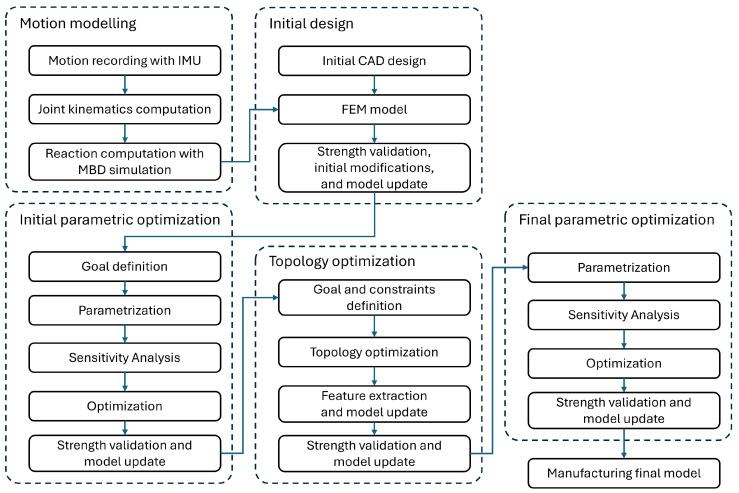
Flow diagram of the full optimization process.

Each specific stage of the optimization is described in detail in the following Section 4, Section 5 and Section 6 of the paper. To improve the clarity and reproducibility of the proposed methodology, the full process flow is presented in Figure 2, while the inputs and outputs of each stage are explicitly defined in Table 2.

## 3. Multibody Analysis

**Figure 3:**
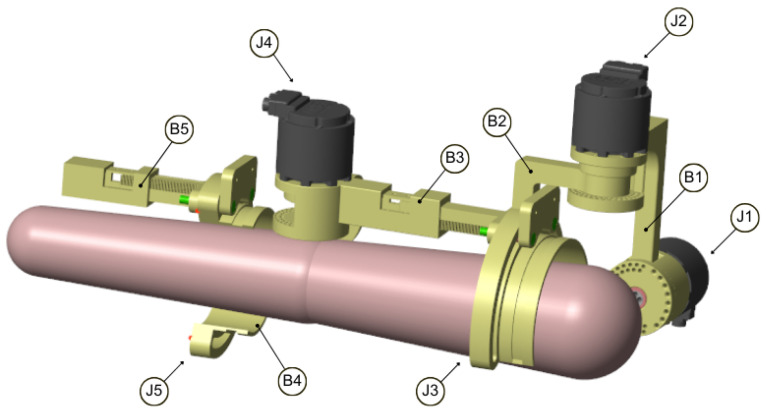
The graphical visual of the used MBS model in its initial configuration with body (B) and joint (J) enumeration.

The rigid multibody simulation (MBS) model enabled dynamic testing based on recorded motion trajectories, resulting in valuable insights into the torque requirements of the motors and internal loads. These insights supported the strength simulations described in Section 4, Section 5 and Section 6. The model was based on real-life measured trajectories and enabled the simulation of the structure’s behavior during motion. Figure 3 shows the MBS model in graphical form within the simulation environment, in its initial configuration—that is, at the start of each recorded trajectory.

The simulation model was primarily developed using the *Simulink* package, along with *Simscape* and *Simscape Multibody*. The exoskeleton bodies used in the simulation were designed in *Autodesk Inventor 2021* and integrated into the *Simscape Multibody* environment. This integration allows for the direct import of 3D models, automatically incorporating physical properties such as density, geometry, and moments of inertia, thus streamlining the model-building process. Moreover, the modified bodies were automatically updated.

The *Robotic System Toolbox* was used to generate and adapt polynomial motion trajectories for the simulation based on the recorded discrete real-life trajectories.

Upon completion of the MBS simulation, the recorded trajectories and other resulting data are exported to the Workspace for further analysis, particularly to identify critical structural loads.

### 3.1. Motion Modeling

Four IMUs from the Movella DOT system were used to record upper extremity motion reflecting activities of daily living (ADLs). Each sensor, recording at 120 Hz, contains a 3-axis gyroscope, accelerometer, and magnetometer, with orientation data delivered as ZYX Euler angles. While most analyzed ADLs primarily involved the upper limb with minimal body motion, one IMU was placed on the trunk to account for natural position changes. Sensors were also placed on the arm and forearm to track the movements of these segments. A fourth IMU was attached to the palm, which was crucial for measuring forearm supination and pronation. This IMU also enabled the recording of wrist motion across two degrees of freedom (radial/ulnar deviation and flexion/extension), allowing precise modeling of the distal characteristic point of the exoskeleton. The attachment scheme is presented in the complementary paper [30].

To ensure consistency, data were recorded following a standardized procedure [30]: (1) performing a heading reset, (2) holding the extremity in a neutral position, (3) conducting ten repetitions of the activity, and (4) transferring the data. In addition to aligning the axes of the IMUs in the same direction, the heading reset minimizes issues related to proper axis recognition. The initialization phase requires the subject to maintain a neutral position for at least 5 s.

Each session included ten repetitions per activity and was completed within five minutes to minimize sensor drift. The recorded data were then transferred from the Movella DOT sensors to mobile storage and stored in a database. A total of 38 recordings, representing 19 ADLs, were selected for further multibody analysis. These recordings captured repeated motions, with each activity performed multiple times using different motion patterns. These ADLs are described in depth in the complementary paper [30]. Further experiments with multiple participants confirmed that the motions included in this investigation cover a wide range of potential user motions.

The stored data were further processed with proprietary scripted software in *MATLAB 2024a*. This resulted in a time series of joint rotations in the MBS model. This procedure consisted of the following steps: (1) loading data and removing measurement value range limits, (2) computing the initial orientations of the IMUs, (3) assuming the default orientation of the body segments’ frames, (4) computing the motion of the body segments’ frames, and (5) computing the rotation history in the joints, as extensively described in a complementary research paper [30].

### 3.2. Model Construction

This subsection is dedicated to the model description, which is described in detail in a complementary research paper [31].

The input values consist of angular trajectories at the following joints of the structure, defining the planned motion of the system. The output values provide critical insights into the system’s performance and include:

- Required drive torques in the active degrees of freedom to achieve the desired motion.
- Mechanical loads acting on individual components, represented as force and moment vectors reflecting real-life conditions.

The environment was configured to reflect real-world conditions, such as gravity. A common global reference frame was established.

The solid sub-bodies were represented by File Solid” blocks, which imported geometry, material properties, and visuals from files modeled in *Autodesk Inventor 2021*. However, a manual definition of mechanical parameters was also possible. The position of individual mechanical parts in the model was manipulated using “Rigid Transform” blocks, which defined a fixed 3D transformation between two frames of reference.

Rotational degrees of freedom between frames of reference were implemented using *Revolute Joint* blocks. The stiffness and damping coefficients of the internal spring-damper force law were experimentally chosen to ensure realistic motion, accounting for inaccuracies and energy losses due to friction.

Linear adjustments were implemented with the *Prismatic Joint* block. These enabled motion along the Z-axis of the base coordinate system. The *Welded Joint* block was used to restrict movement while providing access to reaction load data, permanently linking the base and subsequent frames of reference. The fixed joint’s mounting point corresponded to the actual attachment location of the upper extremity to the structure.

As part of the project, a pin curve slot mechanism was simulated to accurately determine and measure loads in the free (passive) degrees of freedom. Since none of the available blocks were suitable for simulating passive joints, the mechanism was modeled by applying motion constraints and using a 6-DOF joint. As a result, the required angular trajectory was set.

Rotational degrees of freedom and pin curve slot joints were configured to export the required data during the simulation. For each active DOF, motor torques and constraint forces and torques were recorded as outputs. In passive joints, constraint forces and torques on each shaft were also recorded. In the simulation, maximum torques needed for the motions were considered (as for passive therapy).

### 3.3. Selection of Critical Cases

The selected critical force sets were used in further stages of the project for structural analysis and optimization. The selection was simplified—the critical sets were selected based solely on force and torque magnitudes without considering strain or stress values. Consequently, an appropriate safety factor must be applied to ensure the validity of the assumptions, even in cases where the applied force set could generate higher stresses than those identified as critical.

The analysis consists of independently reviewing the reaction (force and torque) trajectories in the joints for each segment and selecting a critical set of reactions. The critical set for one segment may originate from a different recording than that for another segment. The procedure is divided into the following five steps.

**Figure 4:**
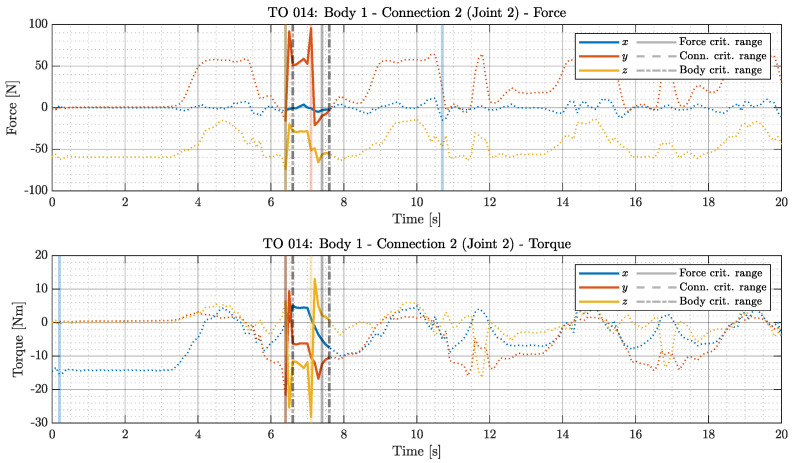
Time history of force and torque in Body 1, Connection 2, Trial 14, and Subject A (part of the total time series).

The first step involves identifying the maximum component values of forces at the joints. The absolute maximum values of the vector components in the coordinate systems related to the joint definitions in the multibody model are recorded. These maxima are marked on an example plot (Figure 4) with vertical solid lines color-coded according to the respective component (blue for x, red for y, and orange for z).

The next step is determining the maximum force and torque norms for each joint. Norm profiles are smoothed using a one-second moving average to eliminate artifacts from recordings and spikes generated in simulations. This smoothing allows identification of a one-second interval where the average norm reaches its maximum value. These intervals are selected independently for forces and torques, for different joints and motions, and are marked on the plot with two solid vertical gray lines (Figure 4).

The selected critical force and torque intervals are normalized to the range $\left[0,\;1\right]$ to remove unit dependency. This facilitates the computation of a new index $p_{Ci}\left(t\right)$ according to Formula (1), where $p_{Ci}\left(t\right)$ represents the normalized, averaged force $F_{Ci}\left(t\right)$, and torque $\tau_{Ci}\left(t\right)$ norm ratio relative to their maximum values (for the *i*-th joint in the given body).

(1) $$p_{Ci}\left(t\right)=F_{Ci}\left(t\right)\cdot \tau_{Ci}\left(t\right)$$

The goal is to determine the time interval during which both forces and torques are simultaneously as close as possible to their peaks. This interval is selected for a particular joint and motion combination (marked on the plot with two vertical dashed gray lines in Figure 4). Ideally, the theoretical maximum appears when $p_{Ci}\left(t^{*}\right)=1$. The MATLAB 2024a script assists in identifying these regions, but a final engineering review is necessary to ensure their validity.

The final processing step selects the critical reaction force set for the entire multibody segment. This involves identifying a time interval during which force and torque norms across all joints within the body are simultaneously near their maximum values. The computation is performed according to Formula (2), where $p_{Bj}\left(t\right)$ represents a new non-physical index for the *j*-th body, while *n* is the number of joints within the body.

(2) $$p_{Bj}\left(t\right)=p_{C1}\left(t\right)\cdot p_{C2}\left(t\right)\cdot \ldots \cdot p_{Cn}\left(t\right),\;\mathrm{for}\;i=1,\;2,\;\ldots ,\;n$$

The interpretation follows that of $p_{Ci}\left(t\right)$ (the identified interval is marked on the plot with a vertical dotted-dashed gray line in Figure 4). Selecting such an interval ensures that the applied forces are consistent and originate from the same time frame, making them representative of actual loading conditions.

Forces and torques are selected to capture the highest possible norm values within the proposed interval to ensure a conservative approach. Consequently, the chosen force value may originate from the beginning of the one-second interval, while the torque value may be taken from its end, independently for each joint in the body.

The final selection of critical forces and torques involves reviewing the average values from the identified intervals (where all joint values come from the same time window) and their corresponding maximum values. Standard deviations were analyzed, and motions with values that deviated significantly (more than one standard deviation) were removed. The goal was to find intervals where the mean remained within the upper boundary of a range spanning two standard deviations and where maximum values were among the highest recorded.

These initially proposed values were further verified by considering intervals for individual joints, forces, and torques separately. The final decision was cross-checked against the force and torque history to eliminate artifacts, measurement errors, or other non-physical simulation results by reviewing the plotted time histories of these values. The outcome—the selected critical forces and torques for each body—is presented in Table 3.

Detailed descriptions of the motion-modeling approach and the multibody model used in this investigation are presented in the complementary papers [30,31].

## 4. Initial Parametric Optimization

### 4.1. FEM Models

To adjust the overall dimensions of the designed exoskeleton, the initial parametric optimization of the robot’s geometric structure was conducted. Five main bodies (assemblies of components connected with joints) were chosen, and their digital models were imported into the *ANSYS 2021 R2* environment. Before any computations, the models were preprocessed to simplify their geometry in order to achieve higher-quality mesh elements in the following stages. This included operations such as simplifying geometry features with negligible influence on the overall durability or splitting bodies with complex structures into smaller, less complex segments with shared topology. Preprocessed models were then transferred to the FEM analysis module.

The materials used for the analyses are similar across the parts and consist of four main linear isotropic material models: aluminum (AlSi10Mg; $E/\rho =26,217,228.5\;m^{2}/s^{2}$), polymer material (SLS-printed nylon; $E/\rho =1,789,473.68\;m^{2}/s^{2}$), structural steel ($E/\rho =25,477,707\;m^{2}/s^{2}$), and brass ($E/\rho =11,547,619\;m^{2}/s^{2}$)—all intended for additive manufacturing. While the material models for brass and steel are part of the *ANSYS Granta Library*, the models of aluminum and polymer material were imported from external libraries. These were assigned to the main optimized components, as they are used in additive manufacturing technologies.

**Figure 5:**
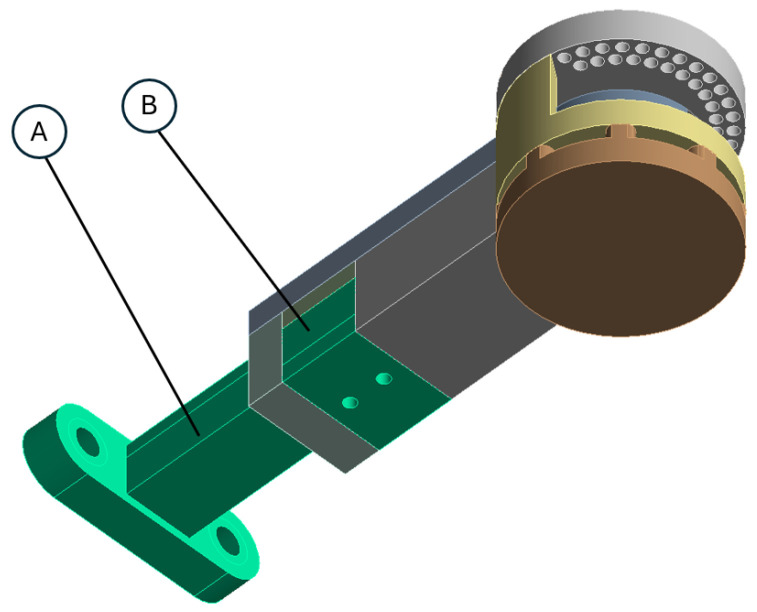
Body 3 regulation part (A) and regulation lock (B).

Each of the analyzed exoskeleton bodies consisted of sub-bodies that were assembled using varying connection types (full assembly is visualized in Figure 3). Interactions between those parts were modeled using contacts. Frictionless contacts were introduced to represent surface-to-surface interaction between main elements (e.g., the regulation part and the regulation lock in Body 3; see Figure 5), while bond contacts were introduced to model bolt connections.

Loads and constraints were applied based on the results derived from Table 3. They are presented in Figures S4–S8. Remote points were introduced to represent points of load application for each body. The methodology used to model boundary conditions is analogous for all optimization stages.

#### Mesh Summary

The mesh grids were generated with the aim of containing hexahedral elements only. The selected mesh density represents a compromise between computational efficiency and numerical accuracy and was verified by preliminary mesh sensitivity checks. Element quality and element skewness were automatically determined by *ANSYS*. The former concerns the comparison of the volumes of the model before and after division. The latter describes how close to equilateral the faces of the created elements are. Their values were used as the main indicators of mesh quality.

The grid of each part was built so that it did not exceed an average element skewness of 0.25 and exceeded an average element quality of 0.80. The resulting mesh parameters are presented in Table 4.

The discrepancies in the nodes-to-elements ratio in Bodies 1 and 5 compared to the other bodies were caused by the usage of the quadratic element order option in *ANSYS*’ mesh tool. Unlike the linear element order, the deformation of a single element is then approximated by a quadratic function, which requires additional nodes in the middle of each of the elements’ edges. Moreover, a higher number of elements within the finite element models of Body 1 and Body 5 resulted from the presence of bolt representations used to attach the motors.

The FEM meshes generated throughout the optimization cycle were verified through a mesh refinement study. This aimed to verify that the FEM results are not significantly affected by the mesh density. Increasing the mesh size by up to 400% did not affect the maximum stress or deformation by more than 8.9% compared with the expected results. This is significantly lower than the assumed safety factors presented in the appendix for the final design. Therefore, the results confirmed that the solution is sufficiently mesh-independent.

### 4.2. Initial Changes to the Design

**Figure 6:**
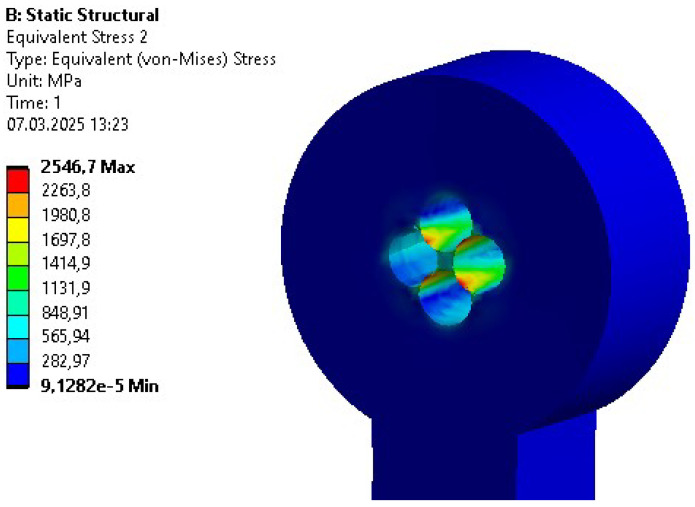
Reduced stress distribution for the bolted connection in the rotational joint.

Initial strength analyses were performed repeatedly due to the variety of explored design adjustments. Similar initial errors were encountered across different bodies. These included four-bolt connections for revolving joints, as shown by the example in Figure 6. The geometry was modified to prevent internal stress from exceeding the material’s ultimate tensile strength.

**Figure 7:**
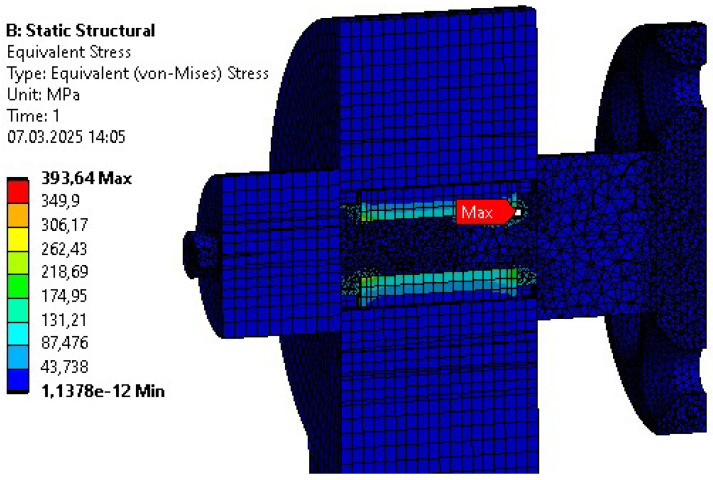
Reduced stress distribution for the key connection in the rotational joint.

Another design utilized a key connection. It was tested during the initial phases of the project. However, the analysis also resulted in excessive stress values in the key and surrounding areas of the optimized part (see Figure 7). Furthermore, due to the size of the shaft, a multi-slot connection was difficult to implement.

The final connection designed and tested for the part was a hexagonal-shaft-based fitted connection. The analysis of this design yielded satisfactory results in terms of stress.

**Figure 8:**
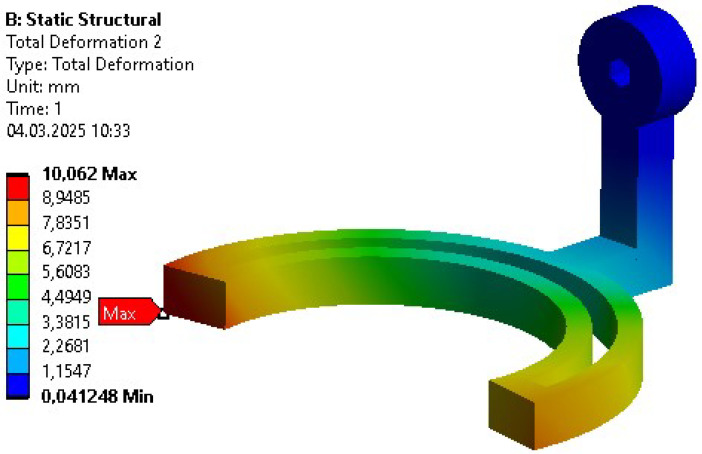
Deformation distribution within a polymer body for the initial design.

Apart from the bodies used for power transmission, the deformation of the overall initial design was also problematic. Only after changing the material from polymer to aluminum was the expected design intent met. Before the material change, a high initial deformation value (above 10 mm) was observed in the model (see Figure 8).

### 4.3. Strength Analysis

The results of the initial strength analysis are presented in Table 5 and Figures S18–S22. As mentioned before, an isotropic aluminum material model was assigned to the main constructional bodies of the exoskeleton. However, some elements, such as sliding bearings, were assigned nylon or brass, and the rotational shafts were assigned structural steel. The analysis was repeated to explore alternative designs and ensure the feasibility of the proposed designs. In general, these maintained satisfactory safety factor values, enabling further reliable parametric modification during the optimization process.

### 4.4. Parametrization

The first parametric optimization step was based on setting dimensional parameters of critical segments of each body. They are illustrated, along with their names and initial values, in Figure S9. Their considered ranges are presented in Table S1.

Most of the ranges were chosen based on constraints from adjacent geometries and overall dimensions. Some of the parameters (e.g., P5) turned out to be more difficult to implement due to their significant impact on the element constraints, while minimally affecting the output parameters. Therefore, they were considered constant for the optimization (their ranges were not presented in Table S1).

### 4.5. Parametric Optimization Method

Parametric optimization was based on a single objective function consisting of the strength analysis output parameters determined beforehand to be the most important in terms of the parts’ feasibility. Function (3) considers not only mass being minimized, but also deformation to guarantee sufficient stiffness of the device, and stress to guarantee sufficient strength. Additionally, optimization constraints regarding maximum deformations and reduced stress are considered. A single goal function comprising three independent parameters was built to exclude human decision-making regarding the selected trade-off during parametric optimization. Therefore, the whole process can be run automatically:

(3) $$f\left(d_{max},m,\sigma_{red_{max}}\right)=a\cdot m+b\cdot d_{max}+c\cdot \sigma_{red_{max}},$$

The parameters in the formula are defined as follows:

- *m*—Geometry Mass
- $d_{max}$—Total Deformation Maximum
- $\sigma_{red_{max}}$—Maximum Stress
- *a*, *b*, *c*—Constant parameters determined for each part individually, based on initial relations between stress, mass, and deformation

The constant parameters selected for optimization may differ depending on the designer’s intentions. The values selected in this investigation are intended to give mass reduction a significant impact while maintaining internal stress and displacement at 10% of the mass impact each. This corresponds to the typical settings of the *OptiSlang* module for such an approach.

Nonlinear Programming by Quadratic Lagrangian Algorithm (NLPQL) is a gradient-based optimization method chosen for the optimization process. Since a global scan of the design space was previously done using sensitivity analysis, there was virtually no risk of convergence to a local minimum, and NLPQL could be chosen as the most suitable method for tackling these particular cases. The desired accuracy of NLPQL was set to 0.005, the differential scheme was set to central, and the differentiation step size was set to 0.001. The maximum number of solver runs was limited to 1000.

Additionally, some constraints were added to the optimization process, such as the minimum acceptable value of the safety factor or correlations between some of the parameters, which ensure the integrity of the design.

### 4.6. Results

The resulting parameters of the initial parametric optimization were assigned to the design and validated through repeated static structural analysis. The results of this validation are presented in Table 6 and Figures S23–S27. The average values of stress and deformation are much lower than those allowed for the design. Moreover, throughout the entire volume of the exoskeleton, the safety factor remains above 5.

## 5. Topology Optimization

The exported models were transferred to new projects, and the static structural analysis was repeated. The greatest difference between this and the previous stage was the mesh requirements. This time, tetrahedral elements were prioritized, since they are more suitable for topology optimization (TO). The mesh was also denser, so the optimization tool was allowed to remove material in smaller portions. This made the resulting geometry smoother and more representative of reality.

The raw *.stl* files, regardless of their quality, were not suitable inputs for the static structural analysis. Therefore, to perform validation calculations, the resulting models were used as references when recreating the geometries and extracting the features acquired by the parts, which were then incorporated into the original models.

### 5.1. FEM Models

The models were meshed into smaller elements as described before. The parameters of the obtained results are summarized in Table 7.

One of the main concerns of topology optimization is retaining previously established functionality in the resulting model. Hence, some regions of the parts are excluded from the analysis. In Figure S11, all excluded areas are marked in red, while regions undergoing the process are marked in blue. Some geometric features, such as teeth in the fifth body in Figure S11f, were excluded and treated as separate bodies. Therefore, they were not colored red or blue but remained in the default coloring (gray).

As previously mentioned, most of the excluded regions were dictated by the goal of retaining the original functionality of the parts. Those regions can be seen in Figure S11b,e, where the inner faces of the ring segment were marked because they are in contact with sliding sleeves and have to provide constant support. Since their geometry is necessary for the connection, the slots for the shaft in the upper segment of the first, second, and fourth parts (Figure S11a,b,e) had to be excluded as well.

The main objective of the whole process is to reduce the mass of the segments. However, additional, less prioritized objectives of compliance and maximum acceptable stress were added to the simulations. This ensures that a certain strength threshold is not exceeded by the parts. Another additional constraint regarding manufacturing was added to guarantee the part’s symmetry along the central plane for some regions of the parts, such as the lower round planes in Figure S11b,f. This restricts the possibility of removing half of the existing geometry, which could jeopardize its ultimate functionality due to asymmetric loads. This also assumes that the device can be used for both extremities.

Moreover, as the parts were manufactured using selective laser sintering additive manufacturing technology [35], minimum wall widths of 1.5 mm were required. Such a manufacturing constraint was added to this optimization stage, except that 0.6 mm was accepted at the locking-tooth edges. At later stages, this criterion was also verified during manual post-processing of the design.

After optimization was performed, all results were exported as raw *.stl* files. They were then processed using *SpaceClaim 2021 R2* tools to repair corrupted regions and obtain the results depicted in Figures S12–S17. These were used as model geometries during the creation of subsequent solids.

### 5.2. Extracted Features

The mesh grids resulting from topology optimization formed the basis for extracting new features for the part designs in regions with regular material retention of at least 5 mm in at least one direction. These were added to the designs and modified according to minor functional modifications (including aligning the external surfaces of the bodies in the base position of the exoskeleton or adjusting the designs for the assembly of rotary encoders). These features are described below with a graphical representation compared with the results from Figures S12–S17.

**Figure 9:**
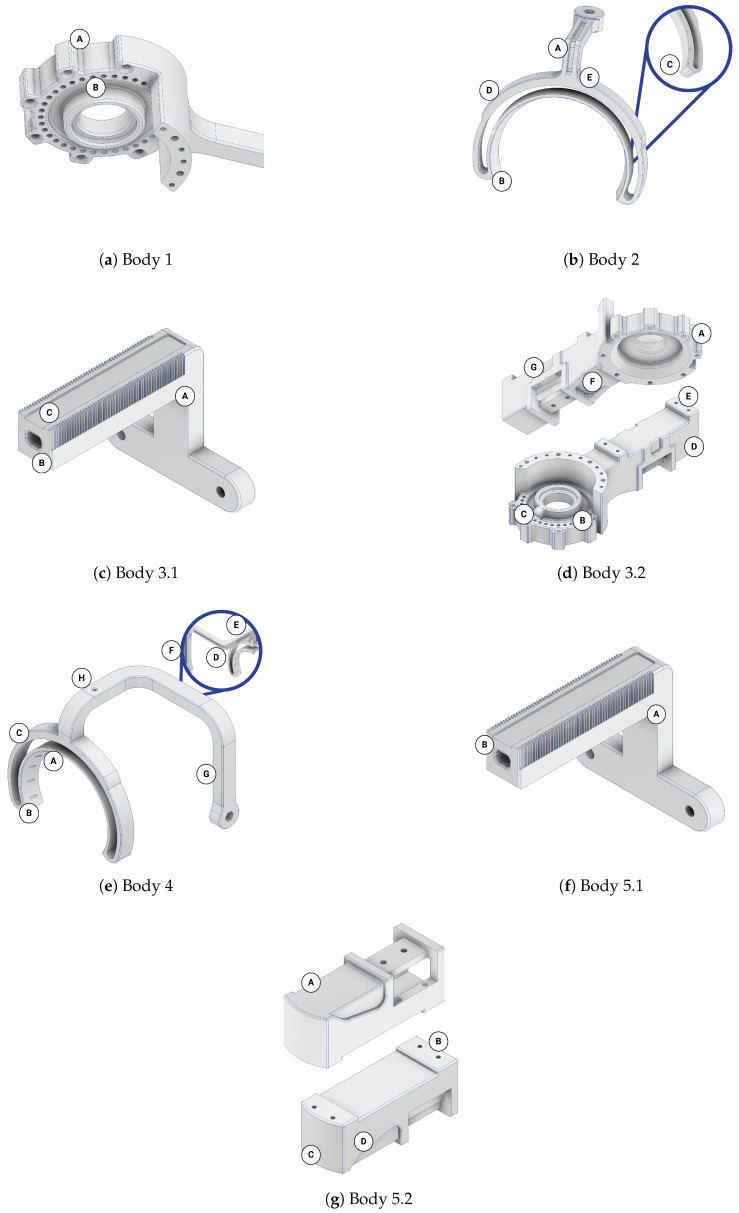
Features extracted from the results of topology optimization.

Body 1 was modified only in the area near the distal joint (see Figure 9a and compare with Figure S12). The joint plate was shaped in a way that reflected the shape of a motor attachment surface **(A)**. Moreover, the inner slot for a bearing was surrounded by the constructional slot **(B)**, reducing mass.

Body 2 had significant modifications in two regions (see Figure 9b and compare with Figure S13). A longitudinal slot **(A)** was placed along the link, and the topology changes were applied in the region of a free-rotation sliding flange. These changes include thinning the internal cylindrical surface **(B)** combined with adding a full-width chamfer **(C)** and removing material from the external area within the set angular range **(D)**. Even though a small material removal appeared in topology optimization next to the connection of the link with the flange (see Figure S13), this feature was omitted in the adjusted design **(E)** as it did not significantly change the mass and would have made the potential design harder to keep clean.

Body 3 was the most modified part of the design. Body 3.1 (see Figure 9c and compare with Figure S14) was changed in shape due to the functional requirement **(A)**. Then, a hole **(B)** was added along its long axis according to the material removal tendency from the optimization. The material reduction from the top face **(C)** was not considered in the feature extraction process, as it could have hindered placing Body 3.1 within a slot of Body 3.2.

Nevertheless, Body 3.2 (see Figure 9d and compare with Figure S15) underwent even more modifications. Like Body 1, its distal joint plate was modified to fit the motor shape **(A)** and to include a cylindrical slot **(B)**. However, it was strengthened with a rib **(C)** in its main plane. The external faces of the sleeve for Body 3.1 were significantly thinned **(D)**—only the areas for attaching the extremity brace **(E)** and two constructional ribs **(F)** next to the blockade slot remained. Furthermore, the placement of the brace was slightly moved to enable the attachment of a three-axis force sensor to detect the intentions of the user for active therapy. Also, rectangular slots **(G)** were added symmetrically on the sides next to the blockade placement.

Analogically, Body 2 and Body 4 (see Figure 9e and compare with Figure S16) had the same modifications in the area of the flange **(A–C)**. However, it had significantly more modifications along the bent link. This resulted in three cavities **(D–F)**, making the segment similar to a thin-wall profile with the full cross-section in the bending corners. One of them **(D)** is a slot starting from one corner and reaching the next bending corner. The other two **(E, F)** were fully closed, as the removal of some mesh elements from the link edges **(G)** was ignored. Nevertheless, technological holes **(H)** were added to enable the metal powder to be emptied after the SLS manufacturing of this component.

In the last part, Body 5 had features similar to those extracted from Body 3. The rod element (Body 5.1, see Figure 9f and compare with Figure S17) was also prolonged and L-shaped **(A)**, and a through-hole **(B)** was added to its main part. The sleeve’s (Body 5.2, see Figure 9g and compare with Figure S17) walls were thinned **(A)**, except for the regions attached to the brace **(B)**, which were also moved. Additionally, the distal face was rounded **(C)**, and additional slots **(D)** were added symmetrically on the sides to minimize the mass.

This stage was critical for adjusting the results of topology optimization for manufacturing while keeping them safe and functional.

### 5.3. Results

The design after feature extraction was validated in terms of its internal stresses, strains, deformation, and the minimum safety factor in the entire structure (see Table 8). The same constraints and loads were applied as in the previous versions, and the design was validated before proceeding to the final optimization. Due to some functionality issues with Body 4 (see Figure S31), its geometry was additionally modified to address these issues, which required adding material in the process. As a result, its mass is slightly higher than in the previous step, despite being subjected to the optimization process.

The design met the predefined success criteria. Therefore, it was subjected to further parametric optimization with a strong focus on the new topologies.

## 6. Final Parametric Optimization

### 6.1. FEM Models

For the final parametric optimization, the mesh grid for each part was generated again. The focus was similar to that of the first parametric optimization—to obtain as many hexahedral elements as possible while maintaining acceptable mesh quality. The latter goal proved problematic since the geometry, previously simple and lacking any distinguishing features, became more complicated and non-smooth. The parameters of the final mesh for each part are presented in Table 9.

### 6.2. Parametrization

Table S2 presents the parameters introduced into the models after the extraction of features. They are depicted in Figure S10. The majority of them are directly connected to these new features, while some are analogous to those from the initial parametric optimization. Nevertheless, dependencies between dimensional and feature-related parameters were also considered.

### 6.3. Results

The whole optimization process was performed with the same methodology (including constraints, contacts, loads, and solver settings) as in the initial parametric optimization. Figures S33–S37 show the resulting deformation and reduced stress distributions in the analyzed parts. Table 10 presents the actual values of the output parameters analyzed in the simulation. In some cases, due to additional functionality requirements, minor modifications were added to the dimensions/topology of the elements. Those changes affected the model masses.

## 7. Discussion

The optimization cycle consisted of three main steps. In all of them, the optimal result was found between 199 and 1000 iterations, which was more correlated to the component’s geometry than to the optimization step. Initial parametric optimization was intended to find generally quasi-optimal dimensions and materials. It resulted in the most significant mass reduction among all of the analyzed bodies. Along with this, it increased the average internal stress, strain, and deformation of the objects. Nevertheless, their values remained significantly below the limits for the selected materials and even increased the minimum safety factor to 4.94. The following topology optimization targeted changes in the components’ dimensions. As a result, the most loaded regions remained solid, while material was removed from the others. This brought further mass reduction. Nevertheless, the safety factor still remained above the acceptable 1.3 threshold and reached a value of 2.6. The last parametric optimization enabled the final small mass reduction by adjusting the parameters of the new features of the design. The other parameters did not vary significantly, and the final safety factor reached a level of 2.52. The total mass of the main constructional bodies decreased by almost 50%, even though the functional modifications that increased the components’ volume were applied before the last parametric optimization. The total mass of the exoskeleton, including motors, mounting components, and sensors, was reduced by 29%.

The results are slightly favorable compared with the single-method optimization presented in the literature for similar devices. Studies typically maintain a safety factor at a level of 1.1–1.33 to achieve a 28–49% mass reduction in constructional elements [23,36,37]. These approaches include only topology optimization or parametric lattice optimization. In contrast, the study that reports the highest safety level (5.9), which is important for medical devices, results in a 43% mass decrease [38].

The optimization results at every stage are presented for separate bodies in Tables S3–S7, while the results for the whole assembly are in Table 11. The columns represent results for the initial design (INITIAL), after initial parametric optimization (PO 1), after topology optimization (TO), and after the second parametric optimization (PO 2), respectively. Internal stress values are presented for the reduced average von Mises stress.

As mentioned above, the first parametric optimization had the greatest contribution to the overall mass reduction. This can be explained by the fact that the initial design contained conservative geometric assumptions, typical for early-stage engineering designs, where safety margins are intentionally high. Parametric optimization enables global scaling of dimensions, which leads to substantial reductions in material volume without fundamentally altering the load paths. The reduction of each individual segment varied from 10% to 50%, while the whole design weight decreased by more than 30% in the complete process. The average values of stress, strain, and deformation increased as expected. Each of these average outputs experienced growth that exceeded 30% of their initial values, with the average deformation almost doubling. The mass reduction was greatest during the initial parametric optimization stage. However, the stages of the complete process affected the parts unevenly. Some benefited greatly, while others experienced only minor changes.

The topology optimization reduced the total mass by a little over 11% compared to its initial value and by 17% compared to the mass from the previous step. The individual mass reduction of each segment varied from −15% to 35% compared to the previous iteration. The negative reduction was caused by the addition of functional features, which required slightly more material than in previous iterations. It is worth noting that the distribution of stress, deformation, and strain, measured by their average values, changed almost as much as after the first parametric optimization.

The final design, determined by the second parametric optimization, reduced the total mass by only about 5% compared to its initial value and by a little under 10% compared to its mass after the topology optimization. Although contributing very little compared to the previous stages, it is important to remember that the lower the mass of the exoskeleton, the harder it is to further optimize it. The highest percentage of mass lost by any individual segment in the entire process is almost 60%, while the lowest is around 40%.

Some of the most vulnerable points in the newly acquired geometries are, naturally, regions around the bends of the links. They are highlighted in the initial strength analysis and remain regions of stress concentration throughout the whole optimization process. Other regions, visible as the most vulnerable across the validation results, are fitted connections to the polygonal shaft of the rotational joints and the bolted connections to the motor mount in Bodies 1 and 3. Moreover, the features introduced based on topology optimization created additional areas of high accumulated stress. Finally, Body 1 has relatively high stress across the whole length of its arm, similar to the newly hollow upper part of Body 4. The increase in stress and deformation observed after successive optimization steps is a natural consequence of mass reduction. As material is removed, structural stiffness decreases, leading to higher stress concentrations and larger displacements. However, all obtained values remained within acceptable safety limits, which confirms the effectiveness of the applied constraints.

The multistep optimization process with hybrid methods enabled significant mass reduction while meeting the strength criteria under real-life loads. However, the presented methods could still be further improved. These could involve different material models, optimization methods, or different orders within the methodology just presented. The parametric optimization process, although supported by sensitivity analysis, might benefit from trying multiobjective optimization to better control the increased maximum values of stress and deformation. Different orders of the optimization types might result in a greater mass reduction or a more even distribution of the reduction between individual stages.

Compared to approaches reported in the literature, the proposed method incorporates realistic loading conditions derived from measured human motion. This represents a significant advantage over studies relying on simplified load cases reflecting operation at the maximum dynamic parameters of the motors, which may lead to overly conservative designs. Moreover, the process guaranteed meeting the defined constraint of a minimum safety factor, particularly important for rehabilitation devices, as they operate in direct physical interaction with humans. Therefore, the presented approach achieves a favorable balance between mass reduction and safety, which is essential for clinical applications.

The design changes reduced its total mass and moments of inertia. These did not significantly affect the device’s stiffness, and the resulting stresses remained within the material’s elastic range. Consequently, the device is expected to operate safely during therapy and should not generate noticeable vibrations. The loads applied in the FEM analysis were derived from the multibody dynamics simulations and represent the most demanding conditions occurring during the rehabilitation motions. Therefore, the obtained stress levels may be considered conservative with respect to the device’s repeated operation. Since the calculated stresses remain well below the material strength limits, no immediate risk from cyclic loading is expected during typical use. Nevertheless, a detailed fatigue and long-term cyclic durability analysis was outside the scope of the present study. As rehabilitation devices undergo a large number of operation cycles, this aspect will be addressed in future work.

Despite the advantages, the proposed approach has some limitations. The optimization is based on a predefined set of motion trajectories, which may not fully represent all possible use cases. Additionally, the FEM models assume simplified material behavior, which may differ from the real-world anisotropic properties of additively manufactured parts. Nevertheless, initial numerical tests showed that the results obtained for average isotropic and orthotropic models were generally consistent for the presented case study. Further investigation of possible improvements in these areas will be part of continuing research.

As expected, modifications of the exoskeleton bodies brought a need for additive manufacturing. Within the research project, this was intentional. However, for practical hospital use, SLS-manufactured surfaces have high roughness. Therefore, they may not comply with hygienic standards and can be more difficult to clean after contamination. Moreover, especially in Body 4, the internal cavities brought technological holes that needed to be enclosed before use. The situation is similar in the regions next to the rotation joints—the removals, similar to the shape of the motor mounting plate, hindered the potential enclosure of the joint required by medical-device standards. However, all of the removed aluminum regions can be easily filled with lightweight polymers.

In contrast, material removal in the mentioned regions also introduced a positive impact on the cost of manufacturing. The costs of 3D-printing technologies are strongly correlated with the amount of material used. After the procedure, the cost of laser sintering aluminum is lower than the initial cost of subtractive manufacturing of the design. Moreover, the additive technology design does not require additional technological divisions of the links.

The presented technology can be used to personalize components easily. It focuses on a combination of strength analysis and motion dynamics simulations. Therefore, every component can be directly modified and validated in terms of its strength. Once this is done, it can be manufactured with a process available in many cities without the need to prepare technical documentation for milling. For this reason, the robot designed to support different individuals can be individualized without constantly engaging professional mechanical designers. Moreover, the proposed methodology can be generalized to other human-interactive robotic systems requiring lightweight yet safe structures.

Reducing the mass and inertia of the main construction parts is expected to directly decrease the joint torques that actuators must generate to track a given rehabilitation trajectory. This can improve actuation efficiency and reduce energy consumption for comparable assistance levels [39]. Moreover, studies on upper-body exoskeletons show that lightweight designs are perceived as more comfortable, enabling longer usage times, which are critical for effective neurorehabilitation [40].

The designed elements were manufactured from AlSi10Mg aluminum using the SLS technique. Tests performed on sintered material samples from the printer used in this study confirmed tensile properties corresponding to those reported in the literature [41]. Therefore, it was assumed that the simulation results reflect the behavior of the real-life parts.

The assembled device was then used for tests of intelligent algorithms controlling rehabilitation exoskeletons with participants. A group of 34 participants underwent experimental trials of active therapy with both intelligent assist-as-needed support and active therapy with constant admittance of the system. The motions performed in the experiments reflected 12 activities of daily living, covering the entire range of the participants’ joint motions and the possible joint velocities assumed to be performed by the system. The exercises were repeated five times each. For comparison, the same exercises were performed manually by the physiotherapist. The total trials took up to 20 min per person for the whole session. The detailed description of these procedures is presented in the complementary research papers. Within the experiments, none of the 3D-printed parts were deformed plastically. Moreover, none of the rotary joint motions was hindered by excessive deformations during the experiments. Considering these results, the design intents of the exoskeleton can be assumed to be met.

## 8. Conclusions

The stated study aims were fulfilled. The mass of the *SmartEx-Home* exoskeleton was minimized through multistep FEM optimization. The procedure presented in this paper consisted of three subsequent optimization stages (parametric, topology, and parametric). Through their application, the dimensional and material parameters were adjusted, new features were extracted, and the final design of the device was established. Using the developed strategy, the total mass of the main components decreased by 49.1% (see comparison in Figure S38).

Combining real-life measured data with digital representations of the modeled objects resulted in lightweight, load-resistant structures for specific applications. This is in line with digital shadow technology (replicating real-life objects as digital models) and the real-life data-driven concept (involving measured human parameters in the engineering process, motions in this case) [42]. The use of such an approach allowed the device design to be durable under expected real-life use cases without overly increasing its mass by assuming excessively high safety factors.

The novelty of the investigation lies in its application to devices that remain in physical contact with humans, based on measured real-life motions. Therefore, the methodology developed is applicable to all robot-aided physiotherapy systems. The methodology of recording real-life motions during interaction with humans, designing multibody models for dynamic simulations, and performing multistep FEM optimization can be used for any device collaborating with a human. These can include physiotherapy robots, assistive devices, or even cobots [43,44,45].

The design of the *SmartEx-Home* exoskeleton, presented as a case study for the developed methodology, was manufactured as part of the research project. It is going to be adjusted for use while attached to everyday objects (e.g., a chair or a bed) or a dedicated movable platform. The exoskeleton will then be tested with human participants in terms of muscular activation during passive and active minimally supervised kinesiotherapy. The functionality of the design has already been verified in terms of motion ranges and adjustment capabilities with a group of ten participants with different anatomies.

## Figures and Tables

**Table 1:** Stages of the complete simulation-based design.

No.	Stage	Purpose
1	Initial design	Creating geometry to compute mass parameters for MBD simulations.
2	Motions modeling	Gathering real-life motions for MBD simulations (control inputs).
3	Inverse dynamics	Computing torques and forces at characteristic points of the model for FEM simulations (loads).
4	Initial parametric optimization	Adjusting overall dimensions and selecting material before topology optimization.
5	Topology optimization	Extracting new features to decrease the mass of the structure.
6	Final parametric optimization	Adjusting dimensions of the extracted features for the final design.

**Table 2:** Overview of the proposed methodology stages (enumeration corresponds to Table 1), with corresponding inputs and outputs.

No.	Input	Output
1	Anthropometric data, functional requirements, previous design’s CAD geometry	Geometric model with mass and inertia properties
2	Predefined rehabilitation tasks	Joint trajectories (position, velocity, acceleration profiles)
3	Joint trajectories, geometry model with mass and inertia properties	Maximal forces and torques at characteristic points
4	CAD geometry, loads from inverse dynamics, material models, boundary conditions, optimization function	FEM model with optimized parameter values and material selection
5	FEM model, design space constraints	Refined CAD geometry with extracted features
6	Refined CAD geometry with extracted features, loads from inverse dynamics, material models, boundary conditions, optimization function	Final optimized geometry with strength parameters: maximum internal reduced stress, maximum displacement, minimum safety factor

**Table 3:** Selected critical forces and torques [t]—connection with the proximal element, [b]—connection with the distal element.

				Force [N]			Torque [N·m]		
Body	Connection	Trial	Subject	x	y	z	x	y	z
1	1	14	A	−68.34	−102.58	5.00	−41.83	26.69	13.15 [t]
	2			−5.00	95.31	−65.70	−7.41	−16.76	−28.13 [b]
2	1	13	B	50.74	−77.85	113.97	28.94	−4.16	15.30 [t]
	2			0.00	0.00	0.00	0.00	−14.42	0.00
	3			0.00	0.00	0.00	−24.01	0.00	0.00
	4			48.31	−112.69	73.65	5.24	4.15	0.00 [b]
3	1	20	B	−147.43	51.46	20.50	9.37	4.05	0.00 [t]
	2			44.08	−126.28	20.73	4.05	−9.37	0.00
	3			21.39	38.69	−21.97	−1.84	2.41	−1.64
	4			18.01	−43.04	22.35	−2.65	−4.64	−4.87 [b]
4	1	13	B	−27.93	40.68	77.32	1.81	−9.43	5.16 [t]
	2			0.00	0.00	0.00	0.00	−2.41	0.00
	3			0.00	0.00	0.00	1.84	0.00	0.00
	4			−68.09	35.66	23.73	1.55	−0.59	0.00 [b]
5	1	5	A	−51.80	22.42	12.24	0.58	1.08	0.00 [t]
	2			14.03	−32.41	12.21	1.08	−0.58	0.00
	3			21.85	17.44	32.71	−2.10	3.69	1.19 [b]

**Table 4:** Mesh metric—initial division.

Parameter	Body 1	Body 2	Body 3	Body 4	Body 5
Number of Elements	233,864	103,152	472,256	176,331	124,071
Number of Nodes	513,462	85,132	260,157	64,793	230,626
Avg. Element Quality	0.81225	0.912	0.800	0.856	0.799
Avg. Skewness	0.24487	0.148	0.251	0.208	0.226

**Table 5:** Results of the initial strength analyses.

Parameter	Body 1	Body 2	Body 3	Body 4	Body 5
Mass [kg]	0.998	0.786	0.808	0.879	0.436
Max. Deformation [mm]	0.17	0.30	0.36	0.36	0.018
Avg. Deformation [mm]	0.09	0.13	0.12	0.23	0.007
Max. Stress [MPa]	32.43	22.70	57.54	31.14	23.79
Avg. Stress [MPa]	1.25	1.77	1.66	1.79	0.97
Max. Strain [‰]	0.4635	0.3527	0.8339	0.5146	0.6500
Avg. Strain [‰]	0.0181	0.0259	0.0244	0.0264	0.0178
Min. Safety Factor	7.44	11.48	4.50	12.9	6.17

**Table 6:** Output parameters after initial parametric optimization (validation analysis).

Parameter	Body 1	Body 2	Body 3	Body 4	Body 5
Mass [kg]	0.572	0.523	0.740	0.420	0.393
Max. Deformation [mm]	0.78	0.29	0.48	2.82	0.06
Avg. Deformation [mm]	0.39	0.13	0.18	0.55	0.02
Max. Stress [MPa]	39.40	22.57	31.04	52.38	20.47
Avg. Stress [MPa]	2.90	1.86	1.75	6.20	0.85
Max. Strain [‰]	0.6097	0.3543	0.4833	0.7724	0.2998
Avg. Strain [‰]	0.0418	0.0271	0.0261	0.0911	0.0134
Min. Safety Factor	5.24	11.48	8.35	4.94	11.24

**Table 7:** Mesh metrics for topology optimization.

Parameter	Body 1	Body 2	Body 3	Body 4	Body 5
Number of Elements	1,926,246	457,250	1,329,921	785,036	1,034,802
Number of Nodes	363,706	667,675	321,296	1,156,409	216,277
Avg. Element Quality	0.850	0.846	0.841	0.847	0.816
Avg. Skewness	0.210	0.216	0.224	0.214	0.245

**Table 8:** Validation of the geometries resulting from topology optimization.

Parameter	Body 1	Body 2	Body 3	Body 4	Body 5
Mass [kg]	0.469	0.443	0.552	0.483	0.252
Max. Deformation [mm]	1.82	0.69	0.95	1.99	0.15
Avg. Deformation [mm]	0.94	0.28	0.39	0.70	0.07
Max. Stress [MPa]	86.04	36.96	107.55	28.78	17.27
Avg. Stress [MPa]	6.78	3.69	3.67	4.52	0.70
Max. Strain [‰]	2.5290	0.5303	1.8078	0.4113	0.2502
Avg. Strain [‰]	0.1102	0.0540	0.0554	0.0655	0.0113
Min. Safety Factor	2.67	6.22	2.60	7.98	15.0

**Table 9:** Mesh metrics for final parametric optimization.

Parameter	Body 1	Body 2	Body 3	Body 4	Body 5
Number of Elements	254,400	206,301	117,562	228,195	645,907
Number of Nodes	402,625	331,080	426,507	368,397	990,991
Avg. Element Quality	0.801	0.837	0.834	0.840	0.837
Avg. Skewness	0.267	0.231	0.232	0.23	0.227

**Table 10:** Validation of final parametric optimization results.

Parameter	Body 1	Body 2	Body 3	Body 4	Body 5
Mass [kg]	0.402	0.472	0.465	0.451	0.200
Max. Deformation [mm]	1.19	0.59	2.62	2.54	0.37
Avg. Deformation [mm]	0.52	0.26	1.03	1.00	0.15
Max. Stress [MPa]	70.60	49.68	111.06	63.88	37.41
Avg. Stress [MPa]	8.37	2.93	5.65	5.61	1.78
Max. Strain [‰]	1.462	0.801	1.881	0.942	0.535
Avg. Strain [‰]	0.126	0.0433	0.0851	0.0850	0.026
Min. Safety Factor	3.25	5.03	2.52	3.60	6.68

**Table 11:** Exoskeleton summary.

Parameter	INITIAL	PO 1	TO	PO 2
Mass [kg]	3.907	2.648	2.199	1.990
Max. Deformation [mm]	0.36	2.82	1.99	2.62
Avg. Deformation [mm]	0.13	0.25	0.43	0.48
Max. Stress [MPa]	57.54	52.38	107.55	111.06
Avg. Stress [MPa]	1.52	2.59	3.47	3.88
Max. Strain [‰]	0.8339	0.7724	1.8078	1.881
Avg. Strain [‰]	0.0228	0.03811	0.0531	0.0582
Min. Safety Factor	4.50	4.94	2.60	2.52

## Data Availability

Data not presented in the paper refer to medical data and cannot be published along with the paper. The data from the FEM simulations are available upon request.

## Supplementary Materials

The following supporting information can be downloaded at https://www.mdpi.com/article/10.3390/s26061849/s1, Figure S1: Visual of using the exoskeleton in standing position; Figure S2: Visual of using the exoskeleton in sitting position; Figure S3: Visual of using the exoskeleton in lying position; Figure S4: Constraints and loads applied to Body 1; Figure S5: Constraints and loads applied to Body 2; Figure S6: Constraints and loads applied to Body 3; Figure S7: Constraints and loads applied to Body 4; Figure S8: Constraints and loads applied to Body 5; Figure S9: Parameters used for initial parametric optimization; Table S1: Ranges of the parameters used for initial parametric optimization; Figure S10: Parameters used in the final parametric optimization (part 3); Table S2: Considered ranges of parameters used in the final parametric optimization; Figure S11: Exclusion (red) and inclusion (blue) regions for topology optimization; Figure S12: Results of topology optimization for Body 1; Figure S13: Results of topology optimization for Body 2; Figure S14: Results of topology optimization for Body 3.1; Figure S15: Results of topology optimization for Body 3.2; Figure S16: Results of topology optimization for Body 4; Figure S17: Results of topology optimization for Body 5; Figure S18: Results of static analysis for Body 1; Figure S19: Results of static analysis for Body 2; Figure S20: Results of static analysis for Body 3; Figure S21: Results of static analysis for Body 4; Figure S22: Results of static analysis for Body 5; Figure S23: Results after initial parametric optimization of Body 1; Figure S24: Results after initial parametric optimization of Body 2; Figure S25: Results after initial parametric optimization of Body 3; Figure S26: Results after initial parametric optimization of Body 4; Figure S27: Results after initial parametric optimization of Body 5; Figure S28: Validation of topology optimization resultant geometry of Body 1; Figure S29: Validation of topology optimization resultant geometry of Body 2; Figure S30: Validation of topology optimization resultant geometry of Body 3; Figure S31: Validation of topology optimization resultant geometry of Body 4; Figure S32: Validation of topology optimization resultant geometry of Body 5; Figure S33: Results after final parametric optimization of Body 1; Figure S34: Results after final parametric optimization of Body 2; Figure S35: Results after final parametric optimization of Body 3; Figure S36: Results after final parametric optimization of Body 4; Figure S37: Results after final parametric optimization of Body 5; Table S3: Summary of Body 1; Table S4: Summary of Body 2; Table S5: Summary of Body 3; Table S6: Summary of Body 4; Table S7: Summary of Body 5; Figure S38: Comparison of initial design (left) and post-optimization cycle design (right) of the exoskeleton.
