# Supplementary material for: Measurement-Based Optimization of a Lightweight Upper-Extremity Rehabilitation Exoskeleton for Task-Oriented Treatment
Source: Sensors (Basel). 2026 Mar 15;26(6):1849. doi: 10.3390/s26061849 (PMC13030215; doi:10.3390/s26061849)
Supplement: Supplementary file 1 [file sensors-26-01849-s001.zip › sensors-4197614-supplementary.pdf]

Supplementary Materials

S1. Visuals of the Device Used in Different Positions

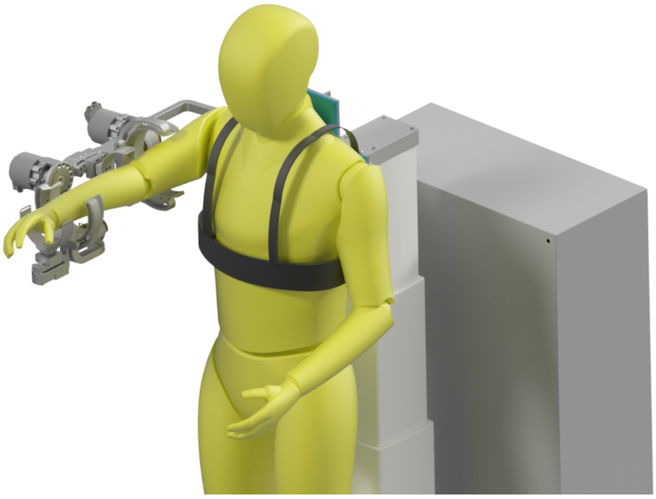

Figure S1. Visual of using the exoskeleton in standing position.

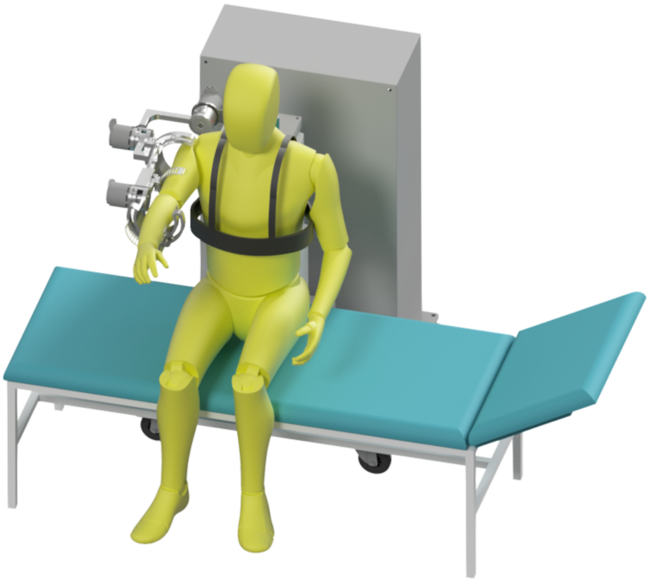

Figure S2. Visual of using the exoskeleton in sitting position.

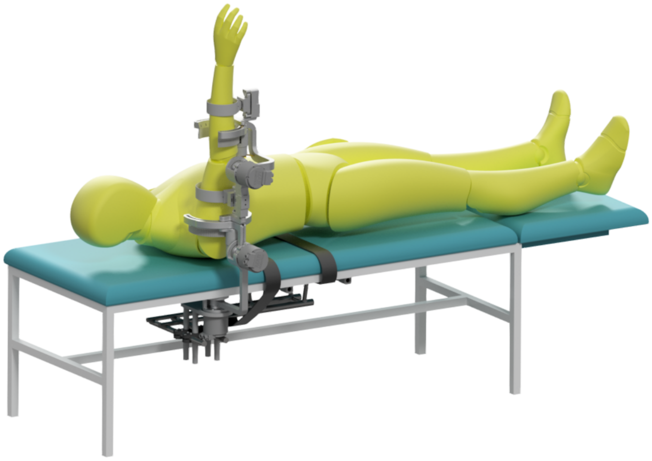

Figure S3. Visual of using the exoskeleton in lying position.

S2. Modeled Connections in the FEM Model

Label	Mechanical connection	Constraint type
A	Face contact with the motor	Compression only
B	Bolted connection to the motor	Compression only
C	Contact with bolt heads	Compression only
D	Contact with sliding sleeve	Cylindrical support
E	Contact with sliding sleeve	Compression only

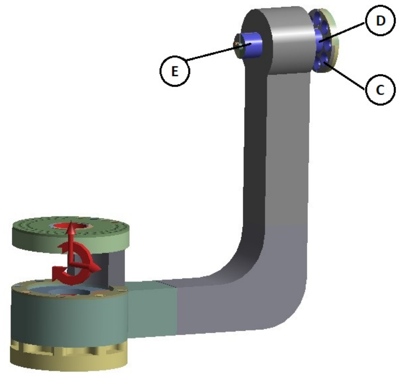

(**a**) Main view

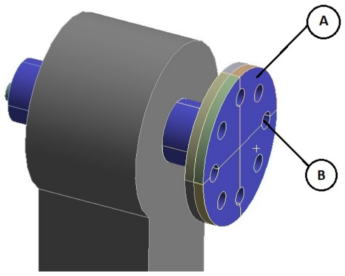

(**b**) Detailed view

Figure S4. Constraints and loads applied to Body 1.

Label	Mechanical connection	Constraint type
A	Contact with sliding sleeve	Compression only
B	Bolted connection to the motor	Cylindrical support
C	Contact with sliding sleeve	Compression only
D	Contact with bolt heads	Compression only
E	Face contact with the motor	Compression only

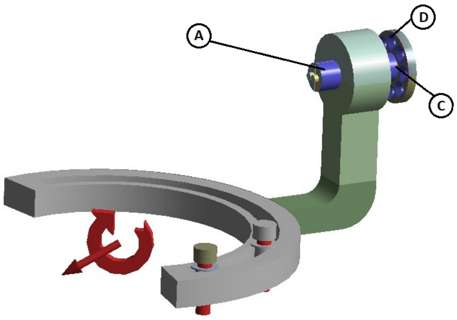

(**a**) Main view

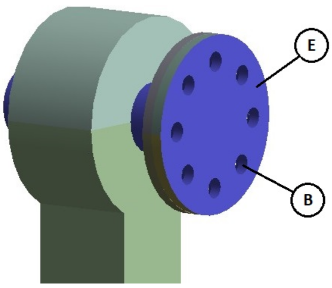

(**b**) Detailed view

Figure S5. Constraints and loads applied to Body 2.

Label	Mechanical connection	Constraint type
A	Contact with sliding sleeve	Compression only
B	Bolted connection to the motor	Cylindrical support

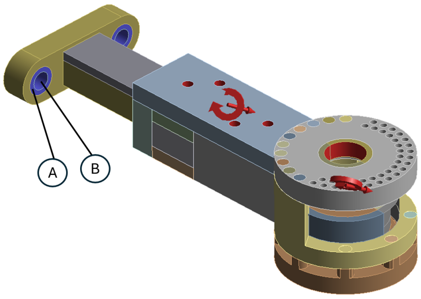

(**a**) Main view

Figure S6. Constraints and loads applied to Body 3.

Label	Mechanical connection	Constraint type
A	Contact with bolt heads	Compression only
B	Contact with sliding sleeve	Compression only
C	Contact with sliding sleeve	Compression only
D	Bolted connection to the motor	Cylindrical support
E	Face contact with the motor	Compression only

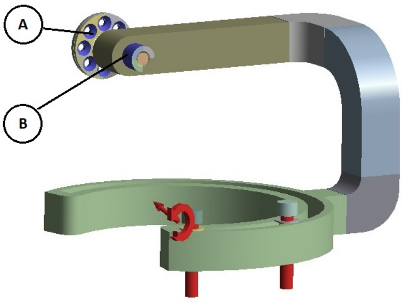

(**a**) Main view

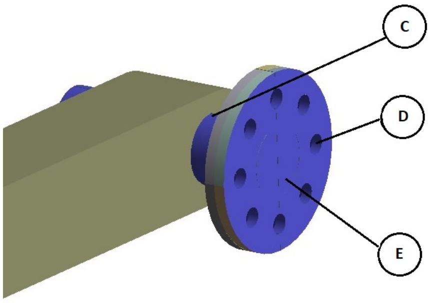

(**b**) Detailed view

Figure S7. Constraints and loads applied to Body 4.

Label	Mechanical connection	Constraint type
A	Contact with sliding sleeve	Compression only
B	Bolted connection to the motor	Cylindrical support

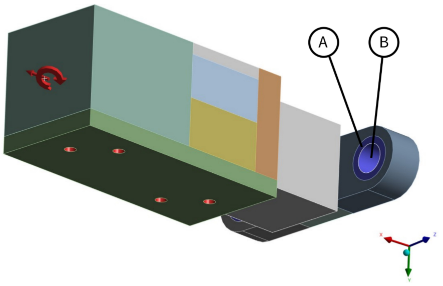

(**a**) Main view

Figure S8. Constraints and loads applied to Body 5.

S3. Parametrization

S3.1. Initial Parametric Optimization

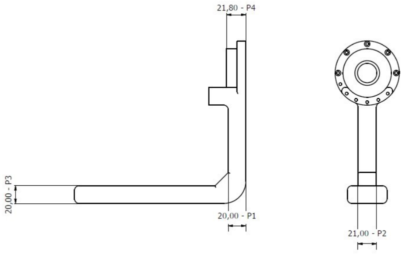

(**a**) Body 1

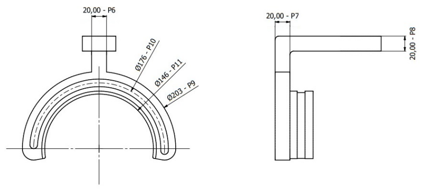

(**b**) Body 2

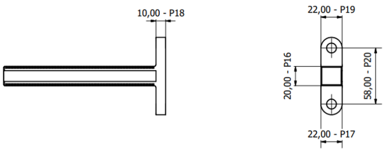

(**c**) Body 3.1

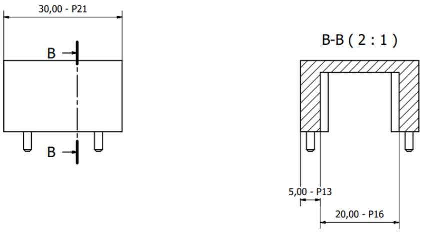

(**d**) Body 3.2

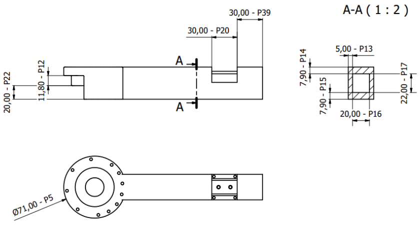

(**e**) Body 3.3

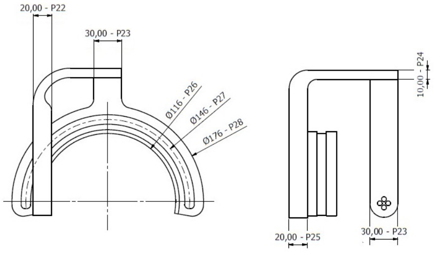

(**f**) Body 4

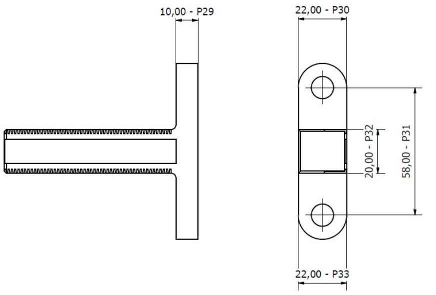

(**g**) Body 5.1

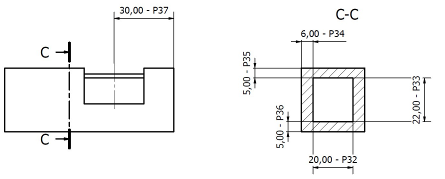

(**h**) Body 5.2

Figure S9. Parameters used for initial parametric optimization.

Table S1. Ranges of the parameters used for initial parametric optimization.

Number	Initial Value [mm]	Minimum [mm]	Maximum [mm]
**Body 1**			
1	20.0	15.0	35.8
2	31.0	20.0	40.0
3	20.0	15.0	30.0
4	11.8	7.8	21.8
5	71.0	-	-
**Body 2**			
6	11.8	7.8	26.8
7	20.0	15.0	40.0
8	20.0	20.0	35.0
9	206.0	180.0	230.0
10	176.0	150.0	190.0
11	146.0	120.0	170.0
**Body 3**			
12	11.8	7.8	21.8
13	5.0	4.0	10.0
14	7.9	5.0	10.0
15	7.9	5.0	10.0
16	20.0	15.0	30.0
17	22.0	15.0	30.0
18	10.0	8.0	15.0
19	22.0	16.0	30.0
20	58.0	-	-
21	30.0	-	-
22	20.0	15.0	30.0
**Body 4**			
22	20.0	15.0	30.0
23	30.0	20.0	30.0
24	10.0	10.0	30.0
25	20.0	18.0	35.0
26	116.0	110.0	140.0
27	146.0	130.0	170.0
28	176.0	160.0	200.0
**Body 5**			
29	10.0	8.0	15.0
30	22.0	15.0	30.0
31	58.0	-	-
32	20.0	15.0	30.0
33	22.0	15.0	30.0
34	6.0	5.0	10.0

S3.2. Final Parametric Optimization

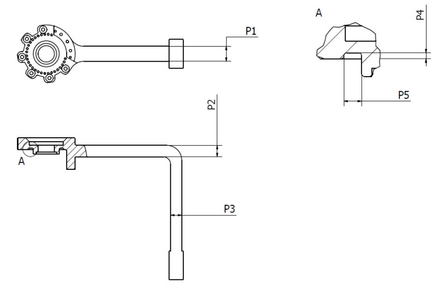

(**a**) Body 1

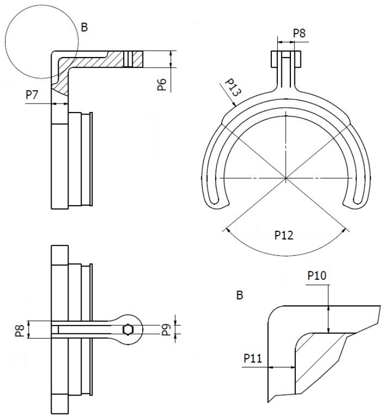

(**b**) Body 2

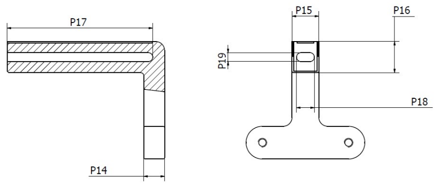

(**c**) Body 3.1

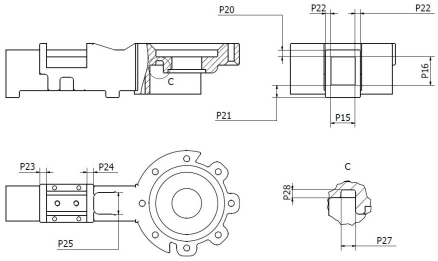

(**d**) Body 3.2

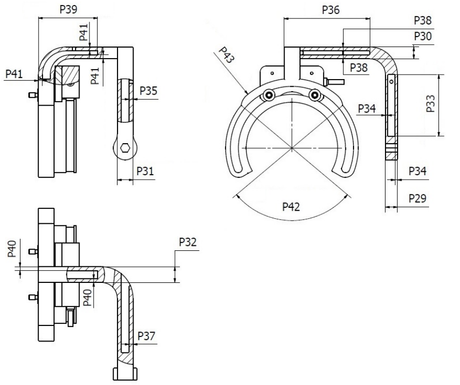

(**e**) Body 4

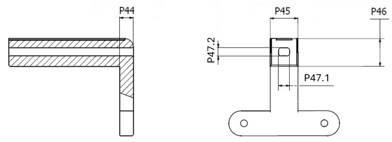

(**f**) Body 5.1

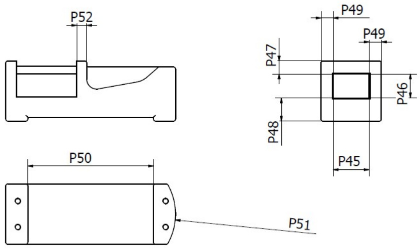

(**g**) Body 5.2

Figure S10. Parameters used in the final parametric optimization (part 3).

Table S2. Considered ranges of parameters used in the final parametric optimization.

Number	Initial Value [mm]	Lower range [mm]	Upper range [mm]
**Body 1**			
1	20.0	10.0	40.0
2	15.0	10.0	40.0
3	15.0	10.0	20.0
4	1.0	0.2	2.0
5	10.0	0.2	24.0
**Body 2**			
6	15.0	10.0	20.0
7	11.0	10.0	20.0
8	20.0	10.0	30.0
9	8.0	0.2	(P8 - 6.0)
10	5.0	0.2	(P6 - 4.0)
11	5.0	0.2	(P7 - 4.0)
**Body 3**			
14	14.	11.0	14.0
15	18.	18.0	63.0
16	21.0	10.0	29.7
17	98.0	0.0	105.0
18	8.0	0.0	(P15 - 10.0)
19	6.0	0.0	(P16 - 10.0)
20	5.0	3.0	(37.7 - P16)/2
21	9.1	8.1	(42.8 - P16)/2
22	5.0	.0	(73.0 - P15)/2
23	5.0	3.0	30.0
24	5.0	3.0	13.0
25	16.0	0.0	(P15 + 2 · P22)
27	5.5	0.0	8.0
28	3.0	0.0	3.0
**Body 4**			
29	20.0	15.0	30.0
30	20.0	15.0	30.0
31	20.0	14.0	30.0
32	20.0	14.0	30.0
33	78.0	22.0	98.0
34	4.0	3.0	(P29/2 - 2.0)
35	5.0	3.0	(P31/2 - 2.0)
36	80.0	45.0	100.0
37	5.0	3.0	(P31/2 - 2.0)
38	4.0	3.0	(P30/2 - 2.0)
39	80.0	40.0	100.0
40	5.0	3.0	(P32/2 - 2.0)
41	4.0	3.0	(P30/2 - 2.0)
42	100.0	30.0	150.0
43	180.0	170.0	200.0
**Body 5**			
44	10.0	8.0	10.0
45	20.0	18.0	63.0
46	20.0	10.0	29.7
47.1	8.0	0.0	(P15 - 10.0)
47.2	6.0	0.0	(P16 - 10.0)
47.3	7.0	6.0	8.0
48	6.0	5.0	7.0
49	5.0	5.0	8.0
50	55.0	0.0	65.0
51	20.0	(P45 + 2 · P49)/2	100.0
52	5.0	3.0	7.0

S4. Topology Optimization

S4.1. Inclusion/Exclusion Regions

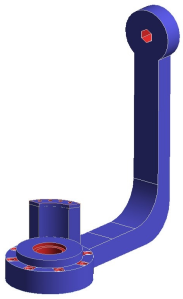

(**a**) Body 1

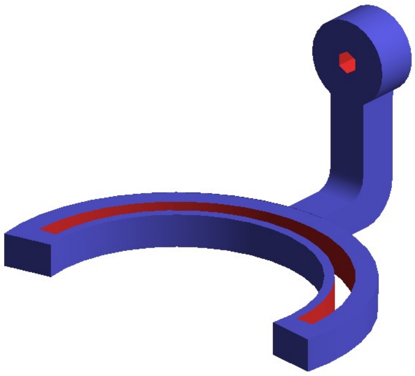

(**b**) Body 2

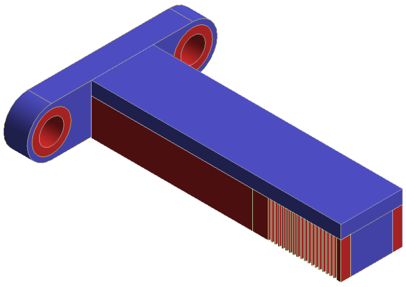

(**c**) Body 3.1

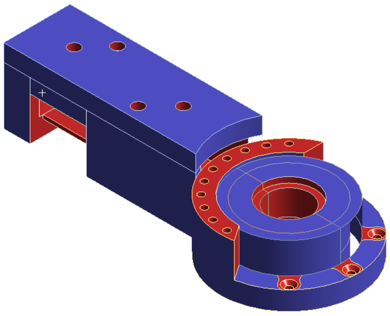

(**d**) Body 3.3

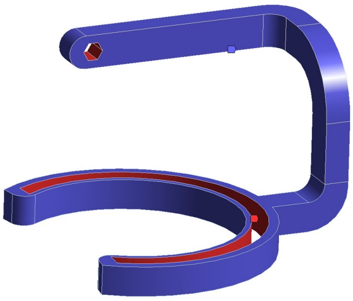

(**e**) Body 4

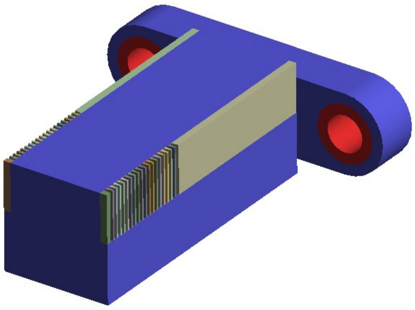

(**f**) Body 5.1

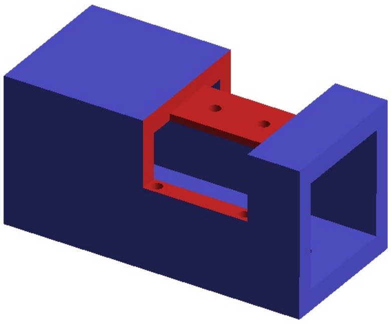

(**g**) Body 5.2

Figure S11. Exclusion (red) and inclusion (blue) regions for topology optimization.

S4.2. Raw Geometrical Results

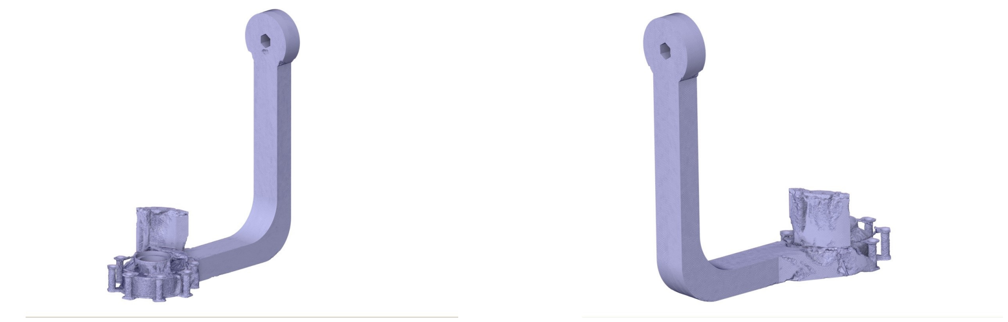

(**a**) Main view (**b**) Secondary view

Figure S12. Results of topology optimization for Body 1.

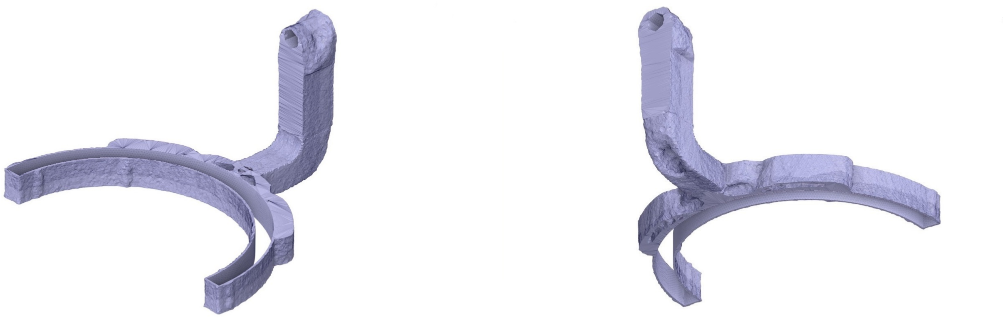

(**a**) Main view (**b**) Secondary view

Figure S13. Results of topology optimization for Body 2.

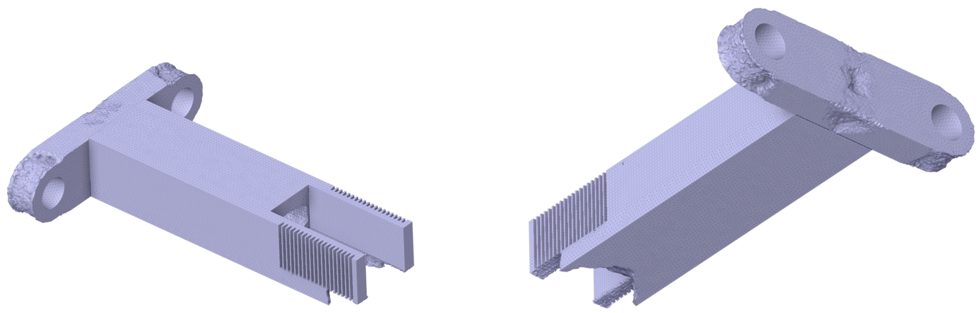

(**a**) Main view (**b**) Secondary view

Figure S14. Results of topology optimization for Body 3.1.

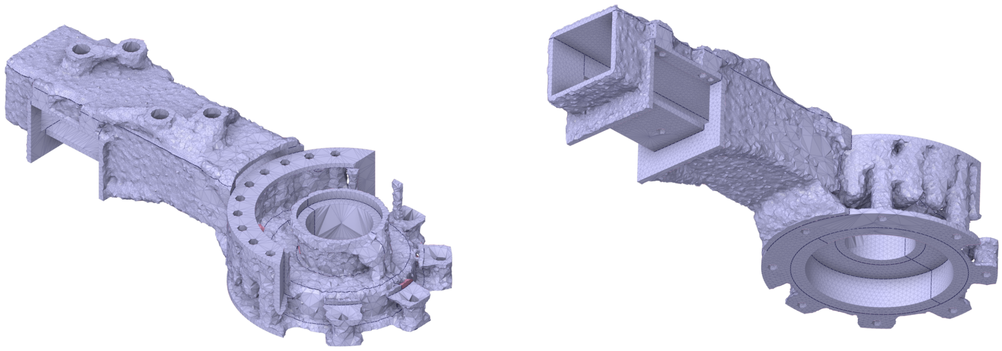

(**a**) Main view (**b**) Secondary view

Figure S15. Results of topology optimization for Body 3.2.

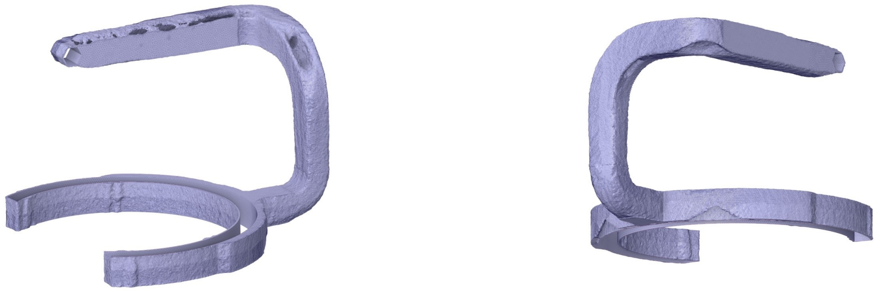

(**a**) Main view (**b**) Secondary view

Figure S16. Results of topology optimization for Body 4.

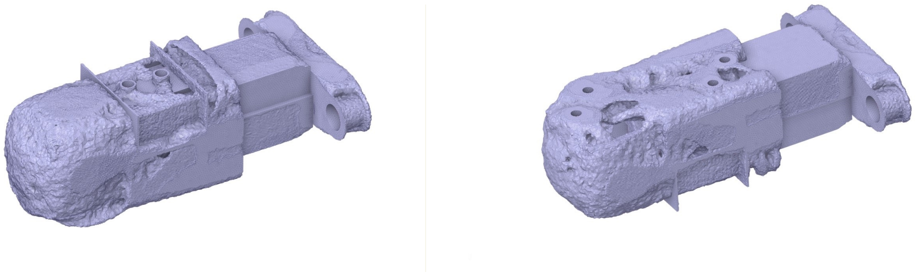

(**a**) Main view (**b**) Secondary view

Figure S17. Results of topology optimization for Body 5.

S5. Results—Deformation and Stress Distribution

S5.1. Initial Strength Analysis

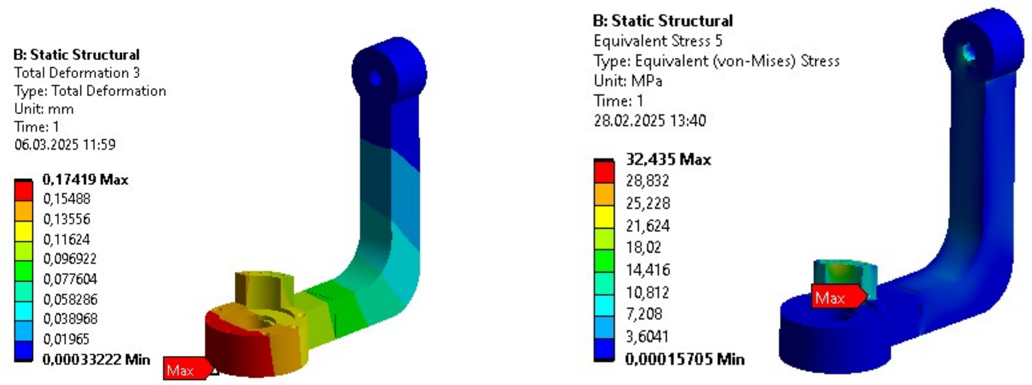

(**a**) Initial deformation distribution (**b**) Initial reduced stress distribution

Figure S18. Results of static analysis for Body 1.

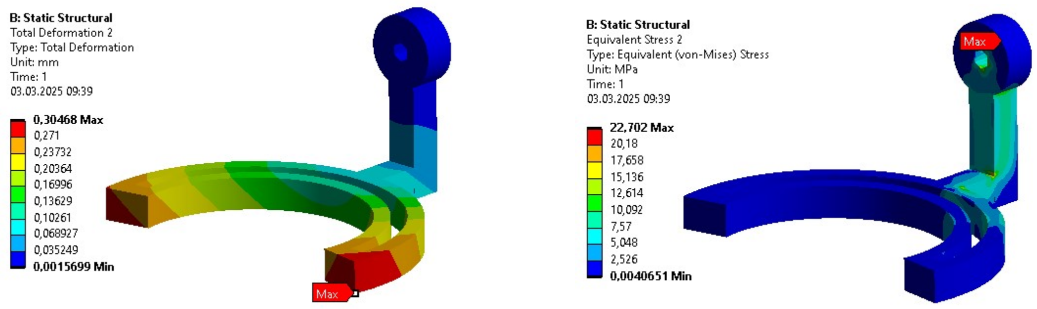

(**a**) Initial deformation distribution (**b**) Initial reduced stress distribution

Figure S19. Results of static analysis for Body 2.

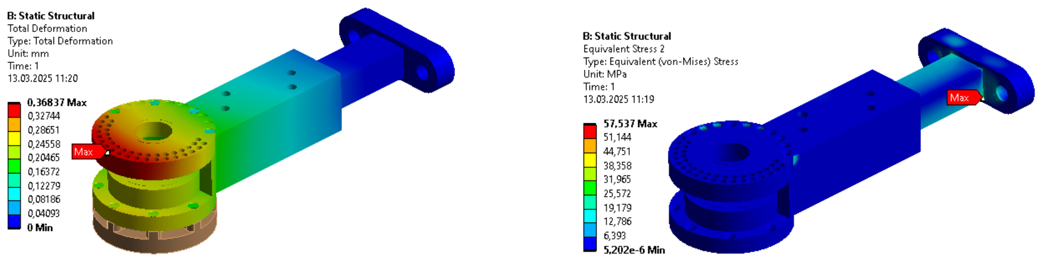

(**a**) Initial deformation distribution (**b**) Initial reduced stress distribution

Figure S20. Results of static analysis for Body 3.

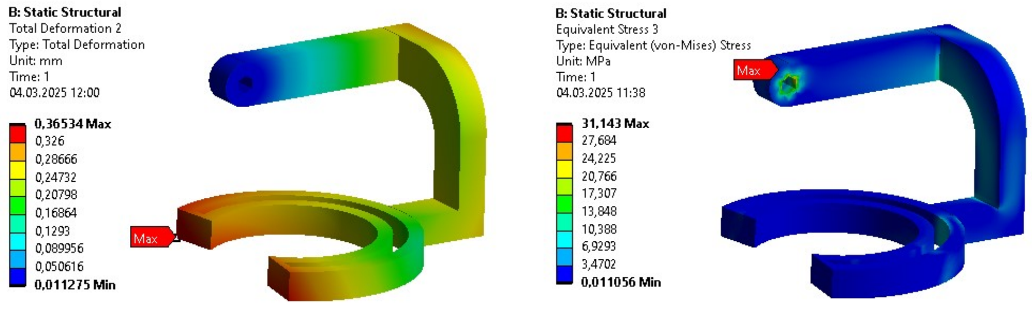

(**a**) Initial deformation distribution (**b**) Initial reduced stress distribution

Figure S21. Results of static analysis for Body 4.

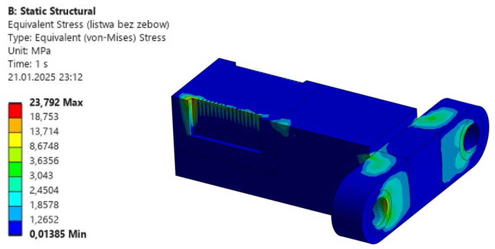

(**a**) Initial reduced stress distribution

Figure S22. Results of static analysis for Body 5.

S5.2. Initial Parametric Optimization

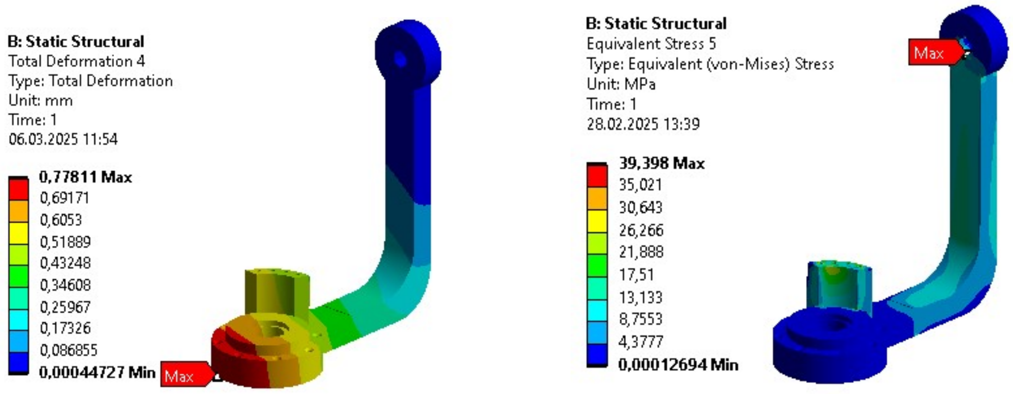

(**a**) Deformation distribution (**b**) Reduced stress distribution

Figure S23. Results after initial parametric optimization of Body 1.

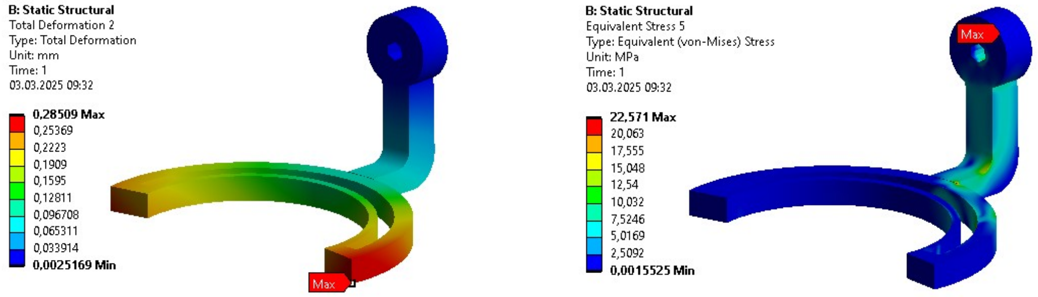

(**a**) Deformation distribution (**b**) Reduced stress distribution

Figure S24. Results after initial parametric optimization of Body 2.

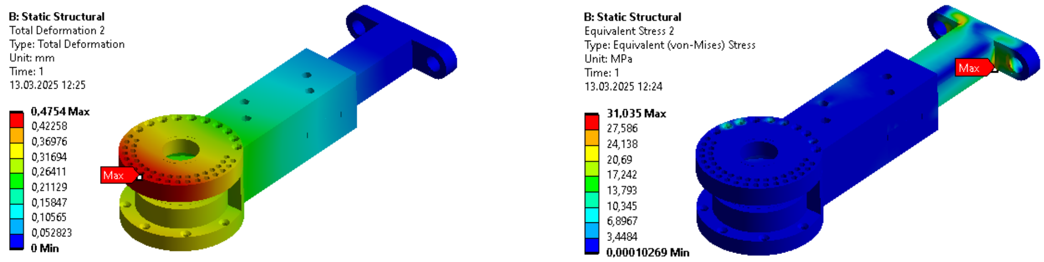

(**a**) Deformation distribution (**b**) Reduced stress distribution

Figure S25. Results after initial parametric optimization of Body 3.

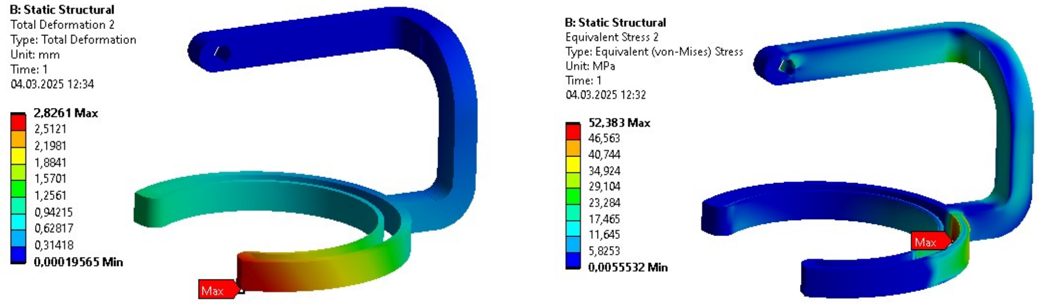

(**a**) Deformation distribution (**b**) Reduced stress distribution

Figure S26. Results after initial parametric optimization of Body 4.

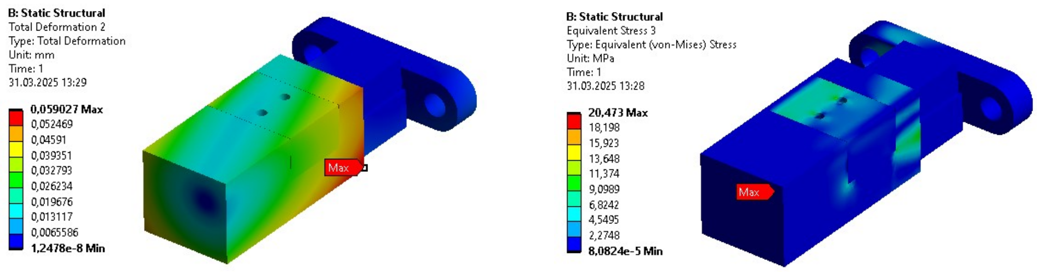

(**a**) Deformation distribution (**b**) Reduced stress distribution

Figure S27. Results after initial parametric optimization of Body 5.

S5.3. Topology Optimization

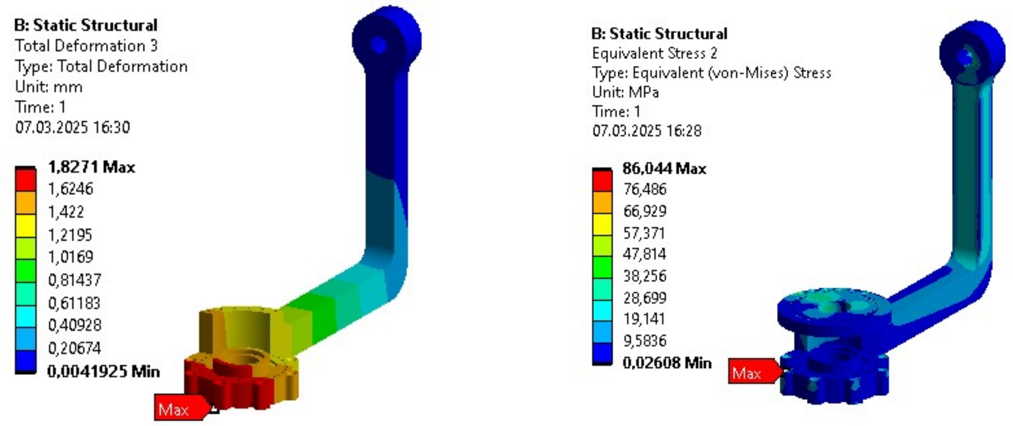

(**a**) Deformation distribution (**b**) Reduced stress distribution

Figure S28. Validation of topology optimization resultant geometry of Body 1.

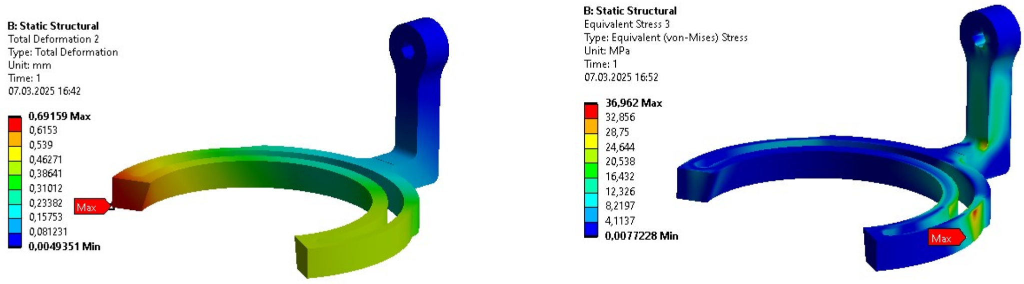

(**a**) Deformation distribution (**b**) Reduced stress distribution

Figure S29. Validation of topology optimization resultant geometry of Body 2.

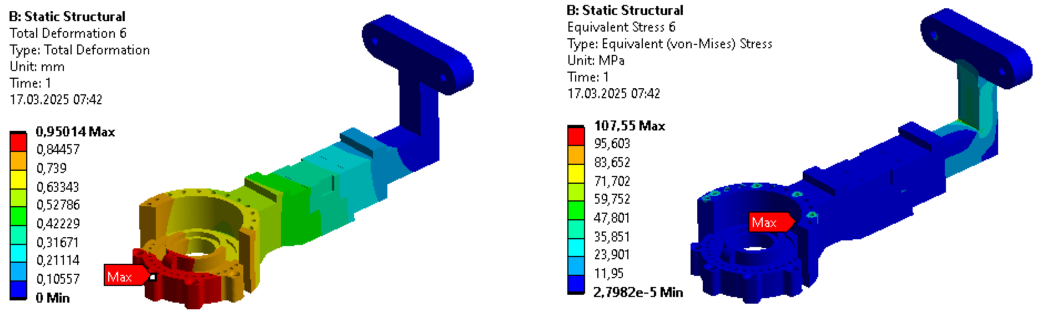

(**a**) Deformation distribution (**b**) Reduced stress distribution

Figure S30. Validation of topology optimization resultant geometry of Body 3.

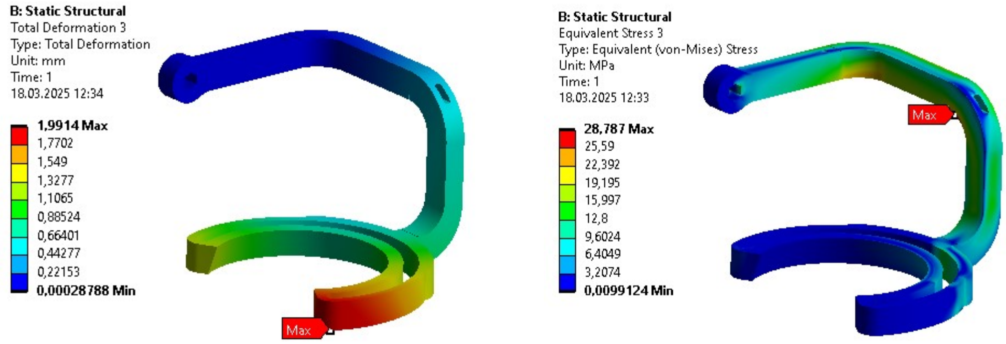

(**a**) Deformation distribution (**b**) Reduced stress distribution

Figure S31. Validation of topology optimization resultant geometry of Body 4.

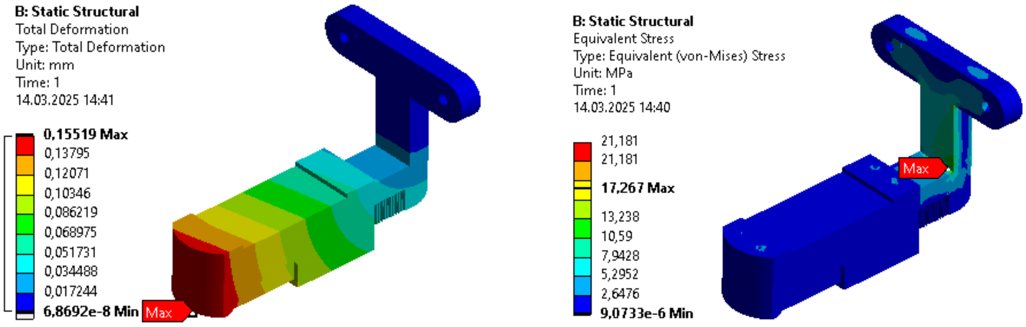

(**a**) Deformation distribution (**b**) Reduced stress distribution

Figure S32. Validation of topology optimization resultant geometry of Body 5.

S5.4. Final Parametric Optimization

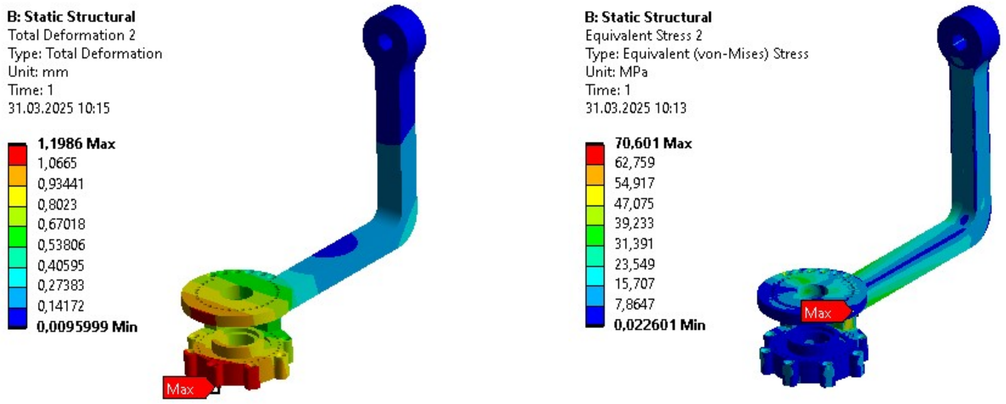

(**a**) Deformation distribution (**b**) Reduced stress distribution

Figure S33. Results after final parametric optimization of Body 1.

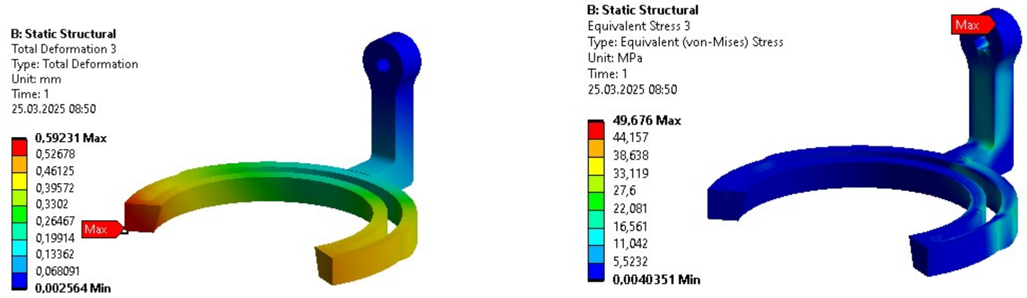

(**a**) Deformation distribution (**b**) Reduced stress distribution

Figure S34. Results after final parametric optimization of Body 2.

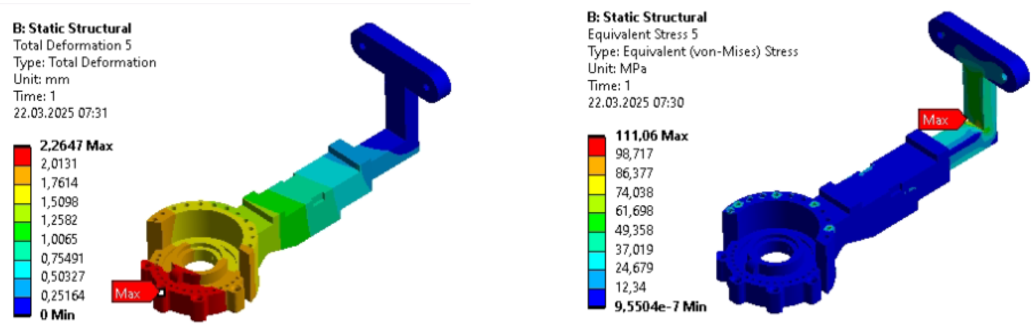

(**a**) Deformation distribution (**b**) Reduced stress distribution

Figure S35. Results after final parametric optimization of Body 3.

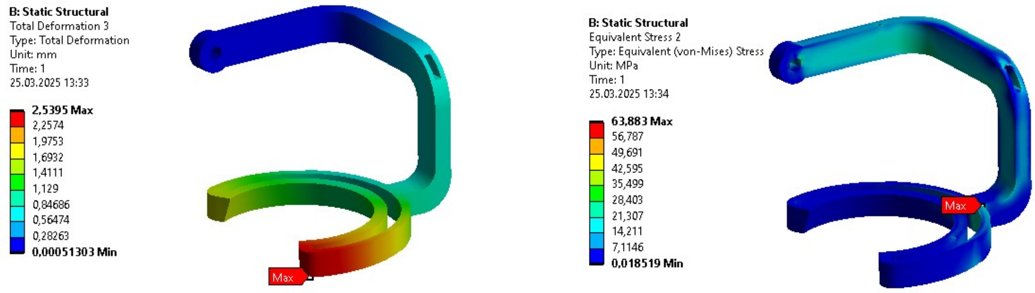

(**a**) Deformation distribution (**b**) Reduced stress distribution

Figure S36. Results after final parametric optimization of Body 4.

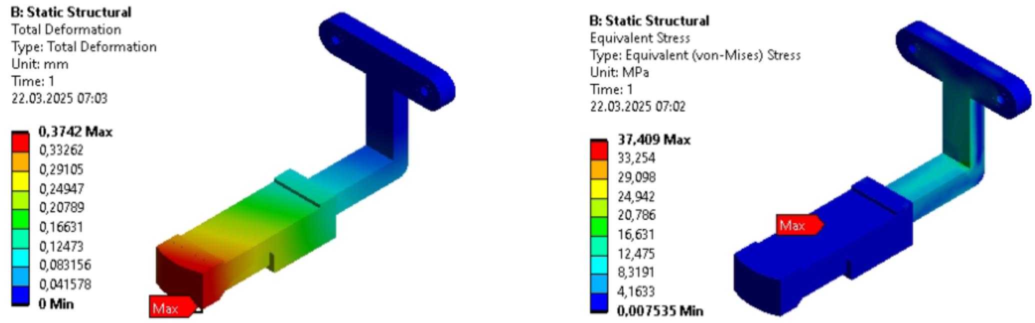

(**a**) Deformation distribution (**b**) Reduced stress distribution

Figure S37. Results after final parametric optimization of Body 5.

S6. Optimization Stages Summary for Each Body

Table S3. Summary of Body 1.

Parameter	INITIAL	PO 1	TO	PO 2
Mass [kg]	0.998	0.572	0.469	0.402
Max. Deformation [mm]	0.17	0.78	1.82	1.19
Avg. Deformation [mm]	0.09	0.39	0.94	0.52
Max. Stress [MPa]	32.43	39.40	86.04	70.60
Avg. Stress [MPa]	1.25	2.90	6.78	8.37
Max. Strain [‰]	0.4635	0.6097	2.529	1.462
Avg. Strain [‰]	0.0181	0.0418	0.1102	0.126
Min. Safety Factor	7.44	5.24	2.67	3.25

Table S4. Summary of Body 2.

Parameter	INITIAL	PO 1	TO	PO 2
Mass [kg]	0.786	0.523	0.443	0.472
Max. Deformation [mm]	0.30	0.29	0.69	0.59
Avg. Deformation [mm]	0.13	0.13	0.28	0.26
Max. Stress [MPa]	22.70	22.57	36.96	49.68
Avg. Stress [MPa]	1.77	1.86	3.69	2.93
Max. Strain [‰]	0.3527	0.3543	0.5303	0.801
Avg. Strain [‰]	0.02591	0.0271	0.0540	0.0433
Min. Safety Factor	11.48	11.48	6.22	5.03

Table S5. Summary of Body 3.

Parameter	INITIAL	PO 1	TO	PO 2
Mass [kg]	0.808	0.740	0.552	0.465
Max. Deformation [mm]	0.36	0.48	0.95	2.62
Avg. Deformation [mm]	0.12	0.18	0.39	1.03
Max. Stress [MPa]	57.54	31.04	107.55	111.06
Avg. Stress [MPa]	1.66	1.75	3.67	5.65
Max. Strain [‰]	0.8339	0.4833	1.8078	1.881
Avg. Strain [‰]	0.02439	0.0261	0.0554	0.0851
Min. Safety Factor	4.50	8.35	2.60	2.52

Table S6. Summary of Body 4.

Parameter	INITIAL	PO 1	TO	PO 2
Mass [kg]	0.879	0.420	0.483	0.451
Max. Deformation [mm]	0.36	2.82	1.99	2.54
Avg. Deformation [mm]	0.23	0.55	0.70	1.00
Max. Stress [MPa]	31.14	52.38	28.78	63.88
Avg. Stress [MPa]	1.79	6.20	4.52	5.61
Max. Strain [‰]	0.5146	0.7724	0.4113	0.942
Avg. Strain [‰]	0.02639	0.0911	0.0655	0.0850
Min. Safety Factor	12.9	4.94	7.98	3.60

Table S7. Summary of Body 5.

Parameter	INITIAL	PO 1	TO	PO 2
Mass [kg]	0.436	0.393	0.252	0.200
Max. Deformation [mm]	0.018	0.06	0.15	0.37
Avg. Deformation [mm]	0.007	0.02	0.07	0.15
Max. Stress [MPa]	23.79	20.47	17.27	37.41
Avg. Stress [MPa]	0.97	0.85	0.70	1.78
Max. Strain [‰]	0.65	0.2998	0.2502	0.535
Avg. Strain [‰]	0.01782	0.0134	0.0113	0.026
Min. Safety Factor	6.17	11.24	15.0	6.68

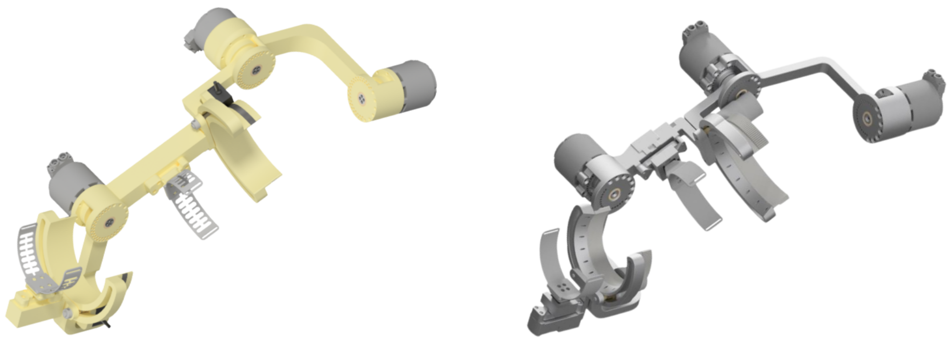

Figure S38. Comparison of initial design (left) and post-optimization cycle design (right) of the exoskeleton.
